# Diagnosis and treatment of acute respiratory illness in children under five in primary care in low-, middle-, and high-income countries: A descriptive FRESH AIR study

**DOI:** 10.1371/journal.pone.0221389

**Published:** 2019-11-06

**Authors:** Jesper Kjærgaard, Marilena Anastasaki, Marianne Stubbe Østergaard, Elvira Isaeva, Azamat Akylbekov, Nhat Quynh Nguyen, Susanne Reventlow, Christos Lionis, Talant Sooronbaev, Le An Pham, Rebecca Nantanda, James W. Stout, Anja Poulsen, FRESH AIR Collaborators

**Affiliations:** 1 Global Health Unit, Department of Paediatrics and Adolescent Medicine, Copenhagen University Hospital “Rigshospitalet”, Copenhagen, Denmark; 2 Clinic of Social and Family Medicine, School of Medicine, University of Crete, Heraklion, Crete, Greece; 3 The Research Unit for General Practice and Section of General Practice, Department of Public Health, Copenhagen University, Copenhagen, Denmark; 4 National Center of Maternity and Childhood Care, Bishkek, Kyrgyzstan; 5 National Center of Cardiology and Internal Medicine, Bishkek, Kyrgyzstan; 6 Family Medicine Department, University of Medicine and Pharmacy, Ho Chi Minh City, Vietnam; 7 Kyrgyz Thoracic Society, Respiratory, Critical Care and Sleep Medicine Department, National Center of Cardiology and Internal Medicine, Bishkek, Kyrgyzstan; 8 Vietnamese Association Family Medicine, Center for training Family Medicine, University of Medicine and Pharmacy, Ho Chi Minh City, Vietnam; 9 Department of Paediatrics, Mulago Hospital and Makere University, Kampala, Uganda; 10 University of Washington, Seattle, Washington, United States of America; Cincinnati Children's Hospital Medical Center, UNITED STATES

## Abstract

**Background:**

Respiratory disease and, specifically, pneumonia, is the major cause of mortality and morbidity in young children. Diagnosis of both pneumonia and asthma in primary care rests principally on clinical signs, history taking, and bronchodilator responsiveness. This study aimed to describe clinical practices in diverse global primary care settings concerning differential diagnosis of respiratory disease in young children, especially between pneumonia and asthma.

**Methods:**

Health professionals in Greece, Kyrgyzstan, Vietnam, and Uganda were observed during consultations with children aged 2–59 months, presenting with cough and/or difficult breathing. Data were analyzed descriptively and included consultation duration, practices, diagnoses and availability/use of medications and equipment. The study is part of the European Horizon 2020 FRESH AIR project.

**Results:**

In total, 771 consultations by 127 health professionals at 74 facilities in the four countries were observed. Consultations were shorter in Vietnam and Uganda (3 to 4 minutes) compared to Greece and Kyrgyzstan (15 to 20 minutes). History taking was most comprehensive in Greece. Clinical examination was more comprehensive in Vietnam and Kyrgyzstan and less in Uganda. Viral upper respiratory tract infections were the most common diagnoses (41.7% to 67%). Pneumonia was diagnosed frequently in Uganda (16.3% of children), and rarely in other countries (0.8% to 2.9%). Asthma diagnosis was rare (0% to 2.8%). Antibiotics were prescribed frequently in all countries (32% to 69%). Short acting β-agonist trials were seldom available and used during consultations in Kyrgyzstan (0%) and Uganda (1.8%), and often in Greece (38.9%) and Vietnam (12.6%).

**Conclusions:**

Duration and comprehensiveness of clinical consultations observed in this study seemed insufficient to guide respiratory diagnosis in young children. Appropriate treatment options may further not be available in certain studied settings. Actions aiming at educating and raising professional awareness, along with developing easy-to-use tools to support diagnosis and a general strengthening of health systems are important goals.

## Introduction

Globally, respiratory disease is among the major causes of morbidity and mortality, and young children are particularly susceptible [1]. Two decades ago, mortality in children under five years old (under-fives) was twice as high as it is today [2]. As a response, the World Health Organization (WHO) launched the Integrated Management of Childhood Illness (IMCI) guidelines [3] as a tool to help health professionals diagnose and treat the main causes of under-five mortality. IMCI has since been implemented in more than 75 countries [4]. The guidelines are designed for use in primary care without sophisticated equipment or skills and a full IMCI assessment takes approximately 8–12 minutes [5].

The main cause of reported mortality in under-fives is pneumonia [2]. Evidence suggests that mortality from lower respiratory diseases other than pneumonia, e.g. asthma, is frequently under-reported [6]. Worldwide, diagnosis of pneumonia in primary care rests primarily on clinical signs [7,8] while diagnosis of asthma mainly depends on history taking and bronchodilator responsiveness [9]. IMCI uses simple clinical signs, such as cough and fast breathing, to diagnose pneumonia [10]. Empiric antibiotic treatment is recommended if specific criteria are met. This has led to a concern that pneumonia is over-diagnosed and antibiotics are prescribed unnecessarily, since IMCI has high sensitivity and low specificity for pneumonia [11]. However, the spectrum of respiratory diseases with overlapping symptoms in young children ranges from acute self-limiting viral infections to bacterial infections to non-communicable diseases [12].

Asthma is the most common non-communicable disease among children, with a global prevalence of 5.5% to 25% [13]. Cohort studies have shown that asthma symptoms often start in early childhood [14]. Studies in low- and middle-income countries have documented a high prevalence of asthma among under-fives diagnosed with pneumonia as defined by IMCI [15–17]. A study from Uganda indicated that 41% of under-fives admitted with acute respiratory illness symptoms actually suffered from asthma or other bronchospastic diseases [18]. Untreated acute asthma may contribute to treatment failure, prolonged illness and pneumonia-attributed mortality [6]. Besides problems with under-diagnosis, there are challenges concerning availability of equipment and necessary medication to diagnose and treat asthma in low-resource settings [19].

Therefore, it is essential that clinical assessment of children with respiratory symptoms is sufficient to elicit signs and symptoms that can help clinicians differentiate between common lower respiratory conditions, like pneumonia and asthma, requiring urgent treatment, versus self-limiting viral infections for which supportive care is most important.

The purpose of this study was to describe clinical practices of health professionals in primary care settings with diverse socio-economic backgrounds with regard to differential diagnosis of respiratory disease, especially between pneumonia and asthma, for children presenting with respiratory symptoms. Our secondary objective was to evaluate the availability of equipment and medications for asthma at the participating health facilities.

## Materials and methods

**Fig 1:**
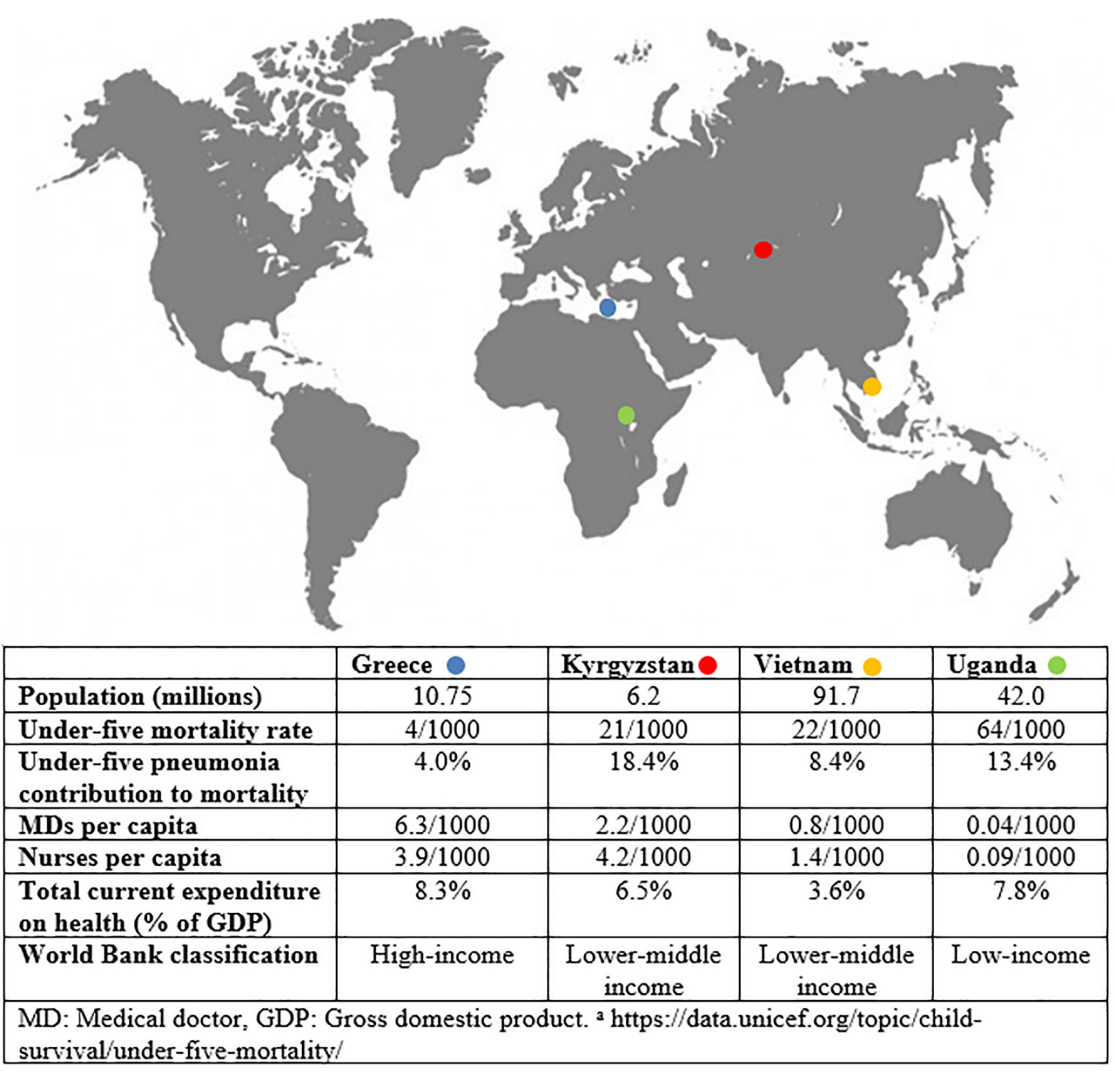
Map of countries participating in the study and key health system indicators.

This descriptive study is part of the European Horizon2020 programme FRESH AIR (https://www.theipcrg.org/freshair) [20]. Data were collected at first line health facilities in Crete, Greece (December 2016-March 2017); Naryn and Chui regions, Kyrgyzstan (April-November 2016); Long An province, Vietnam (December 2016); and Jinja district, Uganda (August-September 2016) (Fig 1). Facilities ranged from referral hospitals also serving as entry point (Greece, Vietnam, Uganda) to health centers (Greece, Kyrgyzstan, Uganda) and single-person manned rural facilities (Kyrgyzstan, Vietnam, Uganda).

Data were collected using three approaches: 1) direct observations of clinical consultations between health professionals and under-fives to document comprehensiveness of consultations, practices and diagnoses, 2) exit interviews with caregivers to obtain background information on the child’s disease and on the present consultation, 3) a short follow-up telephone call with caregivers 5 days after the consultation. This methodology was adapted from WHO’s Health Facility Survey, designed to assess the quality of provided healthcare [21]. We also collected data on human resources, availability of medications and equipment of the participating facilities as detailed in the Health Facility Survey: The health facility administrator filled out a health facility survey form concerning beds, human resources, and medication available in collaboration with the research staff at the first visit to the health facility Research staff then walked through the facility and noted the availability of equipment.

Health professionals were eligible if routine care of children was part of their official duties. They included pediatricians (Greece, Vietnam), general practitioners and nurses (Kyrgyzstan, Vietnam, Uganda), as well as clinical officers with three years of medical training (Kyrgyzstan, Uganda). Children were screened for inclusion at registration desks, triage areas, or waiting lines prior to consultation. Inclusion criteria for children were age of two to 59 months and presenting complaint of cough and/or difficult breathing according to the caregiver.

Upper respiratory tract infections were categorized as “viral infection” if the health professional diagnosed the child with acute respiratory tract infection (ARI), cold, coryza, flu, nasopharyngitis, pharyngitis, respiratory tract infection (RTI), upper respiratory tract infection (URTI), or viral infection. If children received more than one diagnosis, a primary diagnosis was assigned in the following order: asthma, pneumonia, bronchitis, tonsillitis, bronchiolitis, malaria, viral infections. This order was decided by JK and AP taking specificity of symptoms and treatment into account.

All study tools were pilot tested in each country and adapted to fit local contexts. Data were collected by specially trained local staff, including medical doctors, nurses, clinical officers, medical researchers, and public health graduates. Data were typed into REDCap (Research Electronic Data Capture) [22] and exported to STATA Version 15.1 (Stata Corp, Texas, USA) for analysis. Results are summarized using descriptive statistics.

The study was approved by the 7th Health Region of Crete, Hellenic Republic Ministry of Health; Ethics Committee of National Center of Cardiology and Internal Medicine, Bishkek, Kyrgyzstan; IRB committee of University of Medicine and Pharmacy, HCMC, Vietnam; Mulago Hospital Research and Ethics Committee (MREC), the Uganda National Council of Science and Technology, the Leiden University Medical Centre ethical review board, The Netherlands, and The Danish Data Protection Agency (J.nr. 2017-41-5051). All health professionals and caregivers of children signed informed consent forms for participation in the study according to both international and local regulations.

## Results

**Fig 2:**
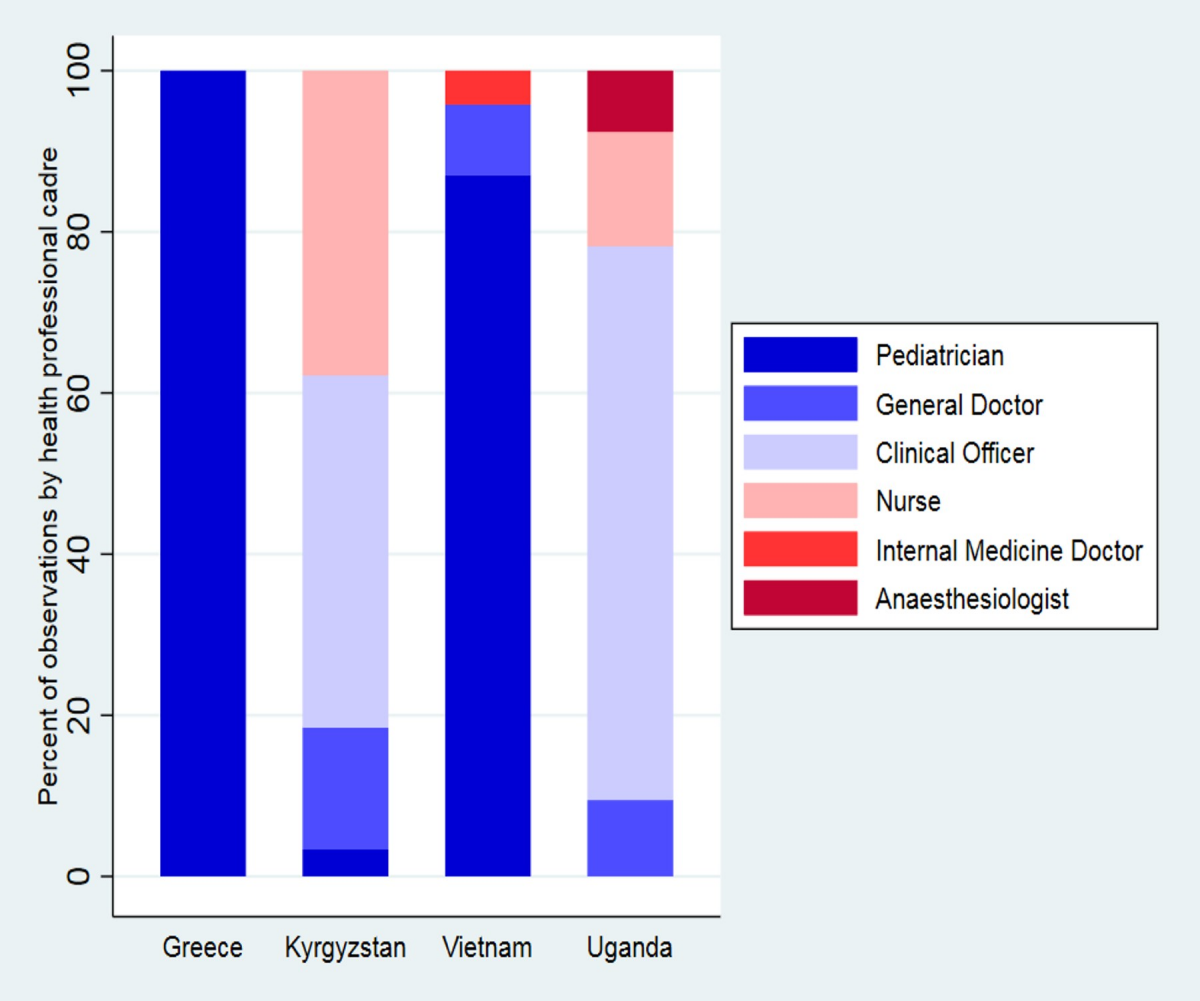
Number of consultations by class of health professional.

In total, 771 clinical consultations made by 127 health professionals at 74 health facilities were observed. The median number of observations per health professional was 4 (IQR 1–7) and the median number of observations performed at each facility was 4 (IQR 2–8). In Kyrgyzstan and Uganda, approximately 80% of consultations were performed by nurses and clinical officers (Table 1, Fig 2).

**Table 1:** Characteristics of health professionals observed during consultations with children aged 2–59 months with respiratory symptoms in low-, middle-, and high-income, primary care settings.

	Greece^a^ n observations = 72	Kyrgyzstan^b^ n observations = 239	Vietnam^c^ n observations = 239	Uganda^d^ n observations = 221	Total N = 771
Number of individual health professionals observed	7	62	9	49	127
Number of observations per health professional, median (IQR)	6 (3–19)	3 (2–5)	12 (10–28)	3 (1–8)	4 (1–7)
**Consultations performed by, n (%)**					
Pediatrician	72 (100)	8 (3.4)	208 (87.0)	0	288 (37.3)
Internal Medicine Doctor	0	0	10 (4.2)	0	10 (1.3)
General Doctor	0	36 (15.1)	21 (8.8)	20 (9.1)	77 (10.0)
Anaesthesiologist	0	0	0	16 (7.2)	16 (2.1)
Clinical Officer	0	104 (43.5)	0	145 (65.6)	249 (32.3)
Nurse	0	90 (37.7)	0	30 (13.6)	120 (15.6)

Information on class of health professional missing for

^a^: 1 observation

^b^: 1 observation

^c^: 0 observations

^d^:10 observations

The proportion of children presenting with respiratory symptoms was 43% to 50% and the duration of symptoms prior to consultation was similar in all countries. Caregivers and children were younger in Uganda and Kyrgyzstan. Approximately 10–15% (Greece, Kyrgyzstan, Uganda) to 40% (Vietnam) of children had five or more previous attendances for respiratory symptoms. In Vietnam, one in four children had 10 or more (Table 2).

**Table 2:** Characteristics of children aged 2–59 months observed during consultations for respiratory symptoms in low-, middle-, and high-income, primary care settings.

	Greece	Kyrgyzstan	Vietnam	Uganda	Total
**Screening and inclusion**					
Number screened for cough and/or difficult breathing, n	166	494	480	486	1626
Number included in study and observed, n (% of screened)	72 (43.4)	239 (48.4)	239 (49.8)	221 (45.5)	771 (47.4)
**Demographic characteristics**					
Maternal age in years, mean (SD)	32·5 (5.8) [1]	29·1 (6.0) [4]	32·3 (6.7) [6]	26·4 (5.1) [9]	29·6 (6.4) [20]
Child age in months, median (IQR)	42 (23.·5–54) [0]	26 (14–38) [0]	31 (19–46) [0]	18 (7–30) [8]	26 (13–42) [8]
Child sex, n female (%)	31 (43.1) [0]	114 (47.7) [0]	106 (44.4) [0]	104 (47.1) [20]	355 (46·0) [20]
**Clinical characteristics**					
Duration of symptoms prior to consultation in days, median (IQR)	3 (2–5) [2]	4 (2–5) [6]	3 (2–5) [95]	3 (2–7) [73]	3 (2–5) [176]
Previous attendance for cough/difficult breathing, n (%)	43 (59.7) [1]	156 (65.3) [4]	186 (77.8) [2]	184 (83.3) [4]	596 (73.8) [11]
Number of previous attendances, n (%):					
1 or 2	28 (38.9)	105 (43.9)	38 (15.9)	53 (24.0)	224 (29.1)
3 or 4	7 (9.7)	29 (12.1)	48 (20.1)	59 (26.7)	143 (18.6)
5 to 9	6 (8.3)	21 (8.8)	34 (14.2)	31 (14.0)	92 (11.9)
10 or more	1 (1.4)	2 (0.8)	57 (23.9)	4 (1.8)	64 (8.3)
No data on previous attendances	30 (41.7)	82 (34.3)	62 (25.9)	74 (33.5)	248 (32.2)
Breathing difficulty at follow-up, n (%)	12 (16.7) [5]	2 (0.8) [8]	12 (5.0) [31]	23 (10.4) [90]	49 (6.7) [134]
Follow-up time in days, median (IQR)	5 (5–5)	5 (5–5)	5 (5–5)	5 (5–5)	5 (4–8)

Numbers in [] represent data that is either missing or question not asked by healthcare professional.

Consultations lasted a median of 5 minutes (IQR 3–20). They were shorter in Vietnam and Uganda (3 to 4 minutes) in contrast to Greece and Kyrgyzstan (15 to 20 minutes). History taking and clinical examination varied greatly. In all countries, several core respiratory symptoms were rarely asked and in Uganda, clinical examination often did not include basic assessment of respiratory disease signs. Questions on former respiratory illnesses were rarely asked by health professionals. Present symptoms typical of asthma, such as difficult breathing, were often asked in Greece (52·8%), but seldom in Kyrgyzstan, Uganda, and Vietnam. Likewise, questions on wheeze or noisy breathing were mostly absent in all countries (Table 3).

**Table 3:** History taking and clinical examination of children aged 2–59 months with respiratory symptoms during consultations in low-, middle-, and high-income, primary care settings.

	Greece n = 72	Kyrgyzstan n = 239	Vietnam n = 239	Uganda n = 221	Total N = 771
Duration of consultation in minutes, median (IQR)	15 (10–30) [14]	20 (20–25) [0]	3 (2–3) [0]	4 (3–4) [4]	5 (3–20) [18]
**Core respiratory symptoms asked, n (%)**					
*History of present illness*					
Fever	63 (87.5) [1]	118 (49.4) [4]	131 (54.8) [0]	75 (34.9) [6]	387 (50.2) [11]
Difficult breathing during this illness	38 (52.8) [1]	36 (15.1) [4]	6 (2.5) [0]	16 (7.2) [7]	96 (12.5) [12]
Wheezing during this illness	6 (8.3) [0]	0 (0) [0]	26 (10.9) [1]	5 (2.3) [7]	37 (4.8) [8]
Noisy breathing	7 (9.7) [1]	10 (4.2) [5]	0 (0) [0]	8 (3.6) [6]	25 (3.2) [12]
Night or early morning cough	25 (34.7) [1]	34 (14.2) [4]	15 (6.3) [0]	15 (6.8) [7]	89 (11.5) [12]
*Past medical history*					
Recurrent difficult breathing	12 (16.7) [1]	0 (0) [5]	0 (0) [0]	5 (2.3) [7]	17 (2.2) [13]
Recurrent cough	33 (45.8) [0]	0 (0) [4]	10 (4.2) [0]	13 (5.9) [9]	56 (7.3) [13]
At least one core respiratory symptom, except fever	53 (73.6) [0]	50 (20.9) [4]	48 (20.1) [0]	34 (15.4) [6]	185 (24.0) [11]
History of triggers	5 (6.9) [1]	0 (0) [0]	0 (0) [12]	7 (3.2) [5]	12 (1.6) [18]
Child or family history of asthma and/or allergy	33 (45.8) [1]	106 (44.4) [4]	4 (1.7) [0]	17 (7.7) [3]	160 (20.8) [8]
Previous medications	48 (66.7) [0]	0 (0) [0]	67 (28.0) [0]	44 (19.9) [4]	159 (20.6) [4]
**Clinical examination performed, n (%)**					
Chest exposed	72 (100) [0]	219 (91.6) [4]	222 (92.9) [0]	47 (21.3) [9]	560 (72.6) [13]
Respiratory rate counted	72 (100) [0]	24 (10.0) [6]	60 (25.1) [0]	24 (10.8) [9]	180 (23.3) [15]
Chest wall in-drawing checked for	41 (56.4) [1]	0 (0) [6]	0 (0) [0]	23 (10.4) [16]	64 (8.3) [33]
Stethoscope used	72 (100) [0]	227 (95.0) [4]	219 (91.6) [0]	35 (15.8) [15]	553 (71.7) [19]
Temperature felt or measured	46 (63.9) [0]	100 (41.8) [4]	57 (23.9) [0]	94 (42.5) [8]	297 (38.5) [12]
Referrals to secondary care	5 (6.9) [2]	8 (3.4) [4]	15 (6.3) [5]	22 (10.0) [28]	50 (6.5) [39]

Numbers in [] represent data that is either missing or question not asked by healthcare professional.

**Fig 3:**
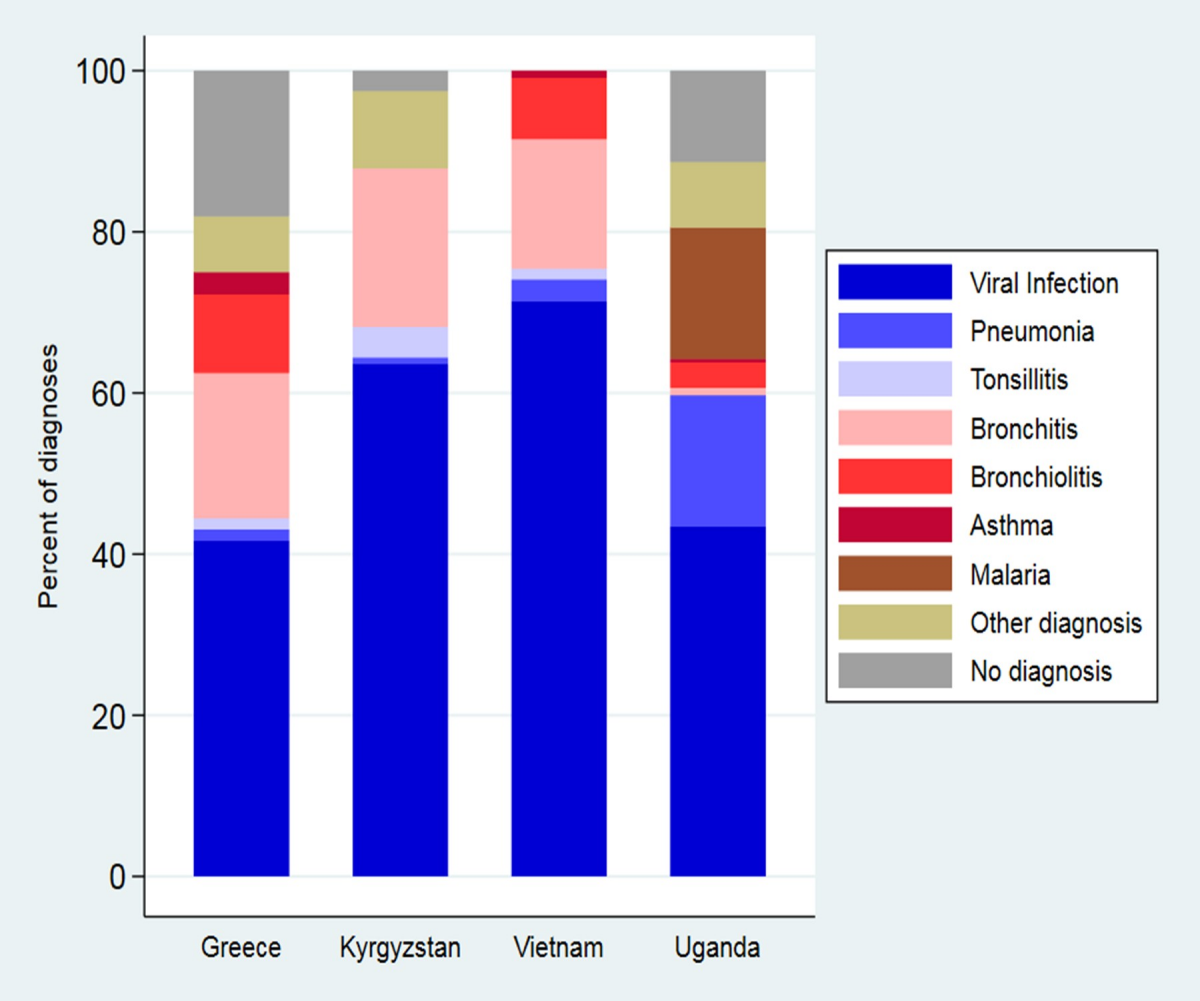
Diagnosis assigned to children aged 2–59 months presenting with respiratory symptoms in low-, middle-, and high-income, primary care settings.

Viral URTIs were most common in all countries, accounting for about half of all diagnoses. Pneumonia was diagnosed frequently in Uganda (16.3%), but rarely in the other countries (0.8% to 2.5%). Bronchitis diagnoses were often used in all countries (15.1% to 19.7%), except Uganda (0.9%). Asthma diagnosis was rare (0% to 2.8%) and malaria was only encountered in Uganda (16.3%) (Table 4, Fig 3).

**Table 4:** Diagnoses and treatments assigned to children aged 2–59 months presenting with respiratory symptoms in low-, middle-, and high-income, primary care settings.

	n (column %)	VURTI^c^	Pneumonia	Tonsillitis	Bronchitis^f^	Bronchiolitis	Asthma	Malaria	Other diagnosis^d^	No diagnosis	Total
**Greece** n = 72	Antibiotics	10 (33.3)	1 (100)	1 (100)	6 (46.2)	-	-	-	2 (40.0)	3 (23.1)	23 (31.9)
	Antivirals and cough medicine^a^	4 (13.3)	-	-	-	-	-	-	-	1 (7.7)	5 (6.9)
	SABA	5 (16.7)	-	-	11 (84.6)	5 (71.4)	2 (100)	-	-	4 (30.8)	27 (37.5)
	Corticosteroids^b^	3 (10.0)	-	-	5 (38.5)	2 (28.6)	-	-	-	3 (23.1)	13 (18.1)
	Supportive treatment^e^	1 (3.3)	-	-	-	-	-	-	1 (20.0)	1 (7.7)	3 (4.2)
	No treatment	8 (26.7)	-	-	-	-	-	-	1 (20.0)	2 (15.4)	11 (15.3)
	***Total (row %)***	***30 (41*.*7) [0]***	***1 (1*.*4) [0]***	***1 (1*.*4) [0]***	***13 (18*.*1) [0]***	***7 (9*.*7) [0]***	***2 (2*.*8) [0]***	***0 (0) [0]***	***5 (6*.*9) [0]***	***13 (18*.*1) [0]***	***72 [0]***
**Kyrgyzstan** n = 239	Antibiotics	80 (52.6)	1 (50.0)	9 (100)	28 (59.6)	-	-	-	16 (69.6)	-	134 (56.1)
	Antivirals and cough medicine^a^	71 (46.7)	-	2 (22.2)	21 (44.7)	-	-	-	6 (26.1)	-	100 (41.8)
	SABA	-	-	-	-	-	-	-	-	-	-
	Corticosteroids^b^	-	-	-	-	-	-	-	-	-	-
	Supportive treatment^e^	-	-	-	-	-	-	-	-	-	-
	No treatment	2 (1.3)	1 (50.0)	-	1 (2.1)	-	-	-	1 (4.4)	1 (16.7)	6 (2.5)
	***Total (row %)***	***152 (63*.*6) [0]***	***2 (0*.*8) [0]***	***9 (3*.*8) [0]***	***28 (19*.*7) [0]***	***0 (0) [0]***	***0 (0) [0]***	***0 (0) [0]***	***23 (9*.*6) [0]***	***6 (2*.*5) [0]***	***239 [0]***
**Vietnam** n = 239	Antibiotics	102 (63.8)	6 (100)	3 (100)	32 (88.9)	12 (70.6)	1 (50.0)	-	-	-	160 (67.0)
	Antivirals and cough medicine^a^	135 (84.4)	3 (50.0)	2 (66.7)	26 (72.2)	11 (64.7)	1 (50.0)	-	-	-	181 (75.7)
	SABA	5 (3.1)	4 (66.7)	1 (33.3)	22 (61.1)	12 (70.6)	1 (50.0)	-	-	-	47 (19.7)
	Corticosteroids^b^	4 (2.5)	1 (16.7)	-	5 (13.9)	3 (17.7)	-	-	-	-	13 (5.4)
	Supportive treatment^e^	-	-	-	-	-	-	-	-	-	-
	No treatment	-	-	-	-	-	-	-	-	-	-
	***Total (row %)***	***160 (67*.*0) [2]***	***6 (2*.*5) [0]***	***3 (1*.*3) [0]***	***36 (15*.*1) [1]***	***17 (7*.*1) [1]***	***2 (0*.*8) [0]***	***0 (0) [0]***	***0 (0) [0]***	***0 (0) [0]***	***239 [12]***
**Uganda** n = 221	Antibiotics	22 (22.9)	14 (38.9)	-	-	4 (57.1)	-	20 (55.6)	5 (50.0)	4 (16.0)	73 (33.0)
	Antivirals and cough medicine^a^	-	-	-	-	-	-	-	-	-	-
	SABA	-	-	-	-	-	-	-	-	-	-
	Corticosteroids^b^	-	-	-	-	-	-	1 (2.8)	-	-	1 (0.5)
	Supportive treatment^e^	44 (45.8)	8 (22.2)	-	-	3 (42.9)	-	9 (25.0)	1 (5.6)	1 (4.0)	66 (29.9)
	No treatment	-	-	-	-	-	-	-	-	-	-
	***Total (row %)***	***96 (43*.*4) [0]***	***36 (16*.*3) [0]***	***0 (0) [0]***	***2 (0*.*9) [0]***	***7 (3*.*2) [0]***	***1 (0*.*5) [0]***	***36 (16*.*3) [0]***	***18 (8*.*1) [0]***	***25 (11*.*3) [0]***	***221 [0]***
**Total** N = 771	Antibiotics	214 (48.9)	22 (48.9)	13 (100)	66 (67.4)	16 (51.6)	1 (20.0)	20 (55.6)	27 (58.7)	7 (15.9)	390 (50.6)
	Antivirals and cough medicine^a^	210 (48.0)	3 (6.7)	4 (30.8)	47 (48.0)	11 (35.5)	1 (20.0)	-	6 (13.0)	1 (2.3)	286 (37.1)
	SABA	10 (2.3)	4 (8.9)	1 (7.7)	33 (33.7)	17 (54.8)	3 (60.0)	-	-	4 (9.1)	74 (9.6)
	Corticosteroids^b^	7 (1.6)	1 (2.2)	-	10 (10.2)	5 (16.1)	-	1 (2.8)	-	3 (6.8)	27 (3.5)
	Supportive treatment^e^	45 (10.3)	8 (17.8)	-	-	3 (9.7)	-	9 (25.0)	2 (4.4)	2 (4.6)	69 (9.0)
	No treatment	10 (2.3)	1 (2.2)	-	1 (1.0)	-	-	-	2 (4.4)	3 (6.8)	17 (2.2)
	***Total (row %)***	***438 (56*.*8) [2]***	***45 (5*.*8) [0]***	***13 (1*.*7) [0]***	***98 (12*.*7) [1]***	***31 (4*.*0) [1]***	***5 (0*.*7) [0]***	***36 (4*.*7) [0]***	***46 (6*.*0) [0]***	***44 (5*.*7) [0]***	***771 [12]***

SABA: Short acting β-agonist, VURTI: Viral upper respiratory tract infection. Numbers in [] represent data that is either missing or question not asked by healthcare professional.

^a^Including cough medicine.

^b^ Inhaled or oral.

^c^ Consisting of children with the following diagnoses according to health professional report: acute respiratory tract infection, cold, coryza, flu, nasopharyngitis, pharyngitis, respiratory tract infection, upper respiratory tract infection, viral infection.

^d^ Other diagnoses: e.g. croup, otitis, scarlet fever, stomatitis aphtosa, tracheitis, diarrhea.

^e^ Supportive treatment: e.g. paracetamol, ibuprofen, antihistamines, fluids, and in Uganda also artemether/lumefantrine, mebendazole, vitamin A.

^f^ Including bronchitis diagnoses with a variety of prefixes, e.g. acute, obstructive, allergic.

Antibiotics were prescribed frequently in all countries (32% to 67%), namely for viral URTIs (22.9% to 63.8% of cases) and bronchitis (46.2% to 88.9%). They were prescribed for 14 out of 36 (Uganda) to one out of one (Greece) cases diagnosed with pneumonia and to all cases of tonsillitis (Table 4).

Short acting β-agonists were often prescribed for children diagnosed with bronchiolitis, bronchitis, and asthma in Greece and Vietnam, but never in Kyrgyzstan and Uganda (Table 4). Short acting β-agonist trials were used in Greece (38.9% of consultations) and Vietnam (12.6%) but almost never in Kyrgyzstan and Uganda. Short acting β-agonist trials had a positive response in approximately half of cases, according to provider report. Inhaled short acting β-agonists were prescribed for two in five children in Greece and for one in five children in Vietnam but never in Kyrgyzstan or Uganda. In Kyrgyzstan and Vietnam, antivirals and cough medicines were prescribed frequently (30.5% and 75.3%) but rarely in Greece and Uganda (Table 5).

**Table 5:** Use and effect of short acting β-agonist trials in children aged 2–59 months presenting with respiratory symptoms in low-, middle-, and high-income, primary care settings.

	Greece, n = 72	Kyrgyzstan, n = 239	Vietnam, n = 239	Uganda^a^, n = 221	Total, N = 771
**SABA trial performed, n (%)**	28 (38.9)	-	30 (12.6)	4 (1.8)	62 (8.0)
**Positive response to SABA trial, n (%)**	18 (64.3)	-	10 (33.3)	1 (25.9)	29 (46.8)

SABA: Short acting β-agonist

^a^: In Uganda, the majority of clinics only had oral SABAs available.

Children were referred to a higher level of care in 3.6% (Kyrgyzstan) to 10% (Uganda) of cases. Cough and/or difficult breathing were still present in 0.8% (Kyrgyzstan) to 17% (Greece) at five days follow-up. In Greece, 9.7% of children had been admitted to hospital at follow-up, 1.7% in Kyrgyzstan, 3.7% in Vietnam, and 2.8% in Uganda. Mortality at follow-up was 0% in Greece, Kyrgyzstan, and Vietnam, and 0.9% in Uganda: One child aged four months presenting with fever, cough and vomiting, diagnosed with a bacterial infection and one child aged 48 months presenting with fever, cough, chest pain and loss of appetite, diagnosed with an ARI. Both children were assigned to home treatment. The chest of the four months old child was not exposed. During the exit interview, the mother reported: “Treatment given is always the same. He normally gets difficult breathing and also wheezes at night and coughs more at night than at day”.

In Greece, Kyrgyzstan, and Uganda, most surveyed facilities were small, with limited personnel and either no, or a few beds for pediatric care. All facilities had electricity, while oxygen and nebulizers were always available in Greece and Vietnam. With a few exceptions, spacers were only available in Greece. In Greece and Vietnam, all facilities had access to nebulized short acting β-agonists. Metered dose inhaler short acting β-agonists were often available in rural clinics in Greece and Kyrgyzstan and at the referral level in Vietnam (Table 6).

**Table 6:** Human resources, amenities, equipment and medications available at participating primary care facilities.

		At pediatric ward			At health facility			Availability of amenities and equipment				Availability of medication				
Country	Level	Beds	Cots	Pediatricians	MDs	COs	Nurses	Electricity	Oxygen	Nebulizers	Spacers	SABA for nebulizer	MDI SABA	Oral SABA	ICS	OCS
Greece	Rural	0	0	1	19	0	0									
	Rural	0	0	1	6	0	1									
	Rural	2	1	1	10	0	3									
	Rural	1	0	1	8	0	2									
Kyrgyzstan	Rural	0	0	0	1	0	5									
	Rural	0	0	0	0	1	0									
	Rural	0	0	0	0	0	1									
	Rural	0	0	0	0	0	3									
	Rural	0	0	0	1	0	3									
	Rural	0	0	0	0	1	0									
	Rural	0	0	0	0	1	1									
	Rural	0	0	0	1	0	5									
	Rural	0	0	0	0	1	0									
	Rural	0	0	1	3	0	5									
	Rural	0	0	0	5	0	10									
	Rural	0	0	0	0	1	1									
	Rural	0	0	0	0	1	1									
	Rural	0	0	0	0	0	1									
	Rural	0	0	0	2	0	3									
	Rural	0	0	0	0	1	0									
	Rural	0	0	0	0	1	1									
	Rural	0	0	0	2	0	4									
	Rural	0	0	0	0	1	1									
	Referral	-	-	-	87	-	270									
	Referral	15	0	2	0	0	6									
Uganda	Rural	3	1	0	2	2	2									
	Rural	2	2	0	2	2	2									
	Rural	2	0	0	2	2	2									
	Rural	6	0	0	1	2	2									
	Rural	0	0	0	0	1	2									
	Rural	4	2	0	2	3	3									
	Referral	50	10	2	1	2	4									-
Vietnam	Rural	35	4	3	0	2	3									
	Referral	105	10	10	11	0	33									

Answers according to color: Always (dark green); Often (green); Sometimes (yellow); Never (dark red); Yes (light green); No (red) MDs: medical doctors, COs: Clinical Officers, SABA: Short acting β-agonist, MDI: Metered dose inhaler, ICS: Inhaled corticosteroids, OCS: Oral corticosteroids

## Discussion

### Main findings

To our knowledge, this is the first study to describe diagnostic and treatment practices related to ARI in under-fives across primary care settings in high-, middle-, and low-income countries with diverse socio-economic and cultural backgrounds. We documented variations in consultation routines, medication availability and frequency of pneumonia diagnoses, along with very low rates of asthma diagnoses and high rates of unnecessary antibiotic prescriptions.

The level of history taking and clinical examination in Kyrgyzstan, Vietnam, and Uganda is comparable to past findings from other low- and middle-income countries in South-east Asia, South America, and Africa, [5,23,24] indicating that there is still much room for improvement in this regard. In a study from Tanzania, longer consultations were associated with better quality of clinical assessment [23]. In Greece and Kyrgyzstan, there was sufficient time for a full IMCI assessment, whereas in Uganda and Vietnam time spent was even shorter than in other low-resource countries.^5^

As expected, most children were diagnosed with viral URTIs, yet different diagnostic patterns were observed across countries. For example, pneumonia was frequently diagnosed in Uganda and rarely in the other countries, while the opposite applied to bronchitis. This may reflect variations in incidence, health seeking behavior, diagnostic criteria, or health professionals’ knowledge or practices. Studies have indicated that the incidence of pneumonia is higher in low-income countries compared to high-income countries [25]. However, it is notable that these estimates were obtained using the WHO criteria for clinical pneumonia, while it has been shown that in low- and middle-income countries, approximately half of children diagnosed with pneumonia using the same criteria, have asthma or other bronchospastic diseases [15, 17].

The IMCI guidelines suggest a short-acting β-agonist trial to distinguish between pneumonia and asthma. In most Ugandan sites, however, neither inhaled short-acting β-agonists nor spacers were available. In Kyrgyzstan, inhaled short acting β-agonists were reported to be available, but spacers were not. It is worth noting that inhaled short acting β-agonists, inhaled corticosteroids, and spacers are on the WHO essential medicines list [26]. Interestingly, in the countries where short acting β-agonist trials were available and used, symptom relief was observed in relatively high rates, however, only a few cases were diagnosed with asthma.

A recent WHO report identifies unnecessary antibiotic prescriptions among the major issues to be addressed in order to combat the global health threat of antimicrobial resistance [27, 28]. In this study, approximately half of children diagnosed with viral URTI were prescribed antibiotics. In the United States, among 5700 children and teenagers presenting with acute URTI, approximately half received un-indicated antibiotics [29]. Data on the global magnitude of unnecessary antibiotic prescriptions seem scarce [28]. However, the problem is likely even more pronounced in countries where over the counter sale of antibiotics is not regulated, an issue not addressed in our study. Qualitative data from Kyrgyzstan and Uganda show that caregivers often go directly to the pharmacy for repeated episodes of illness with similar symptoms to buy the medicine prescribed on the first visit, to save time and money needed for another consultation (Nantanda *et al*. manuscript in preparation, authors’ unpublished data). Thus, an initial inappropriate prescription of antibiotics can snowball into further unnecessary use, as well as an expectation among caregivers of antibiotic treatment also for viral infections.

Finally, an interesting country-specific finding concerned the use of other antiviral and cough medications. Most children in Vietnam and many in Kyrgyzstan, were prescribed mucolytic agents, cough medicines, or antiviral drugs, despite the fact that there is no sufficient evidence of any effect, particularly in younger children [30] and that such medicines are not generally recommended due to serious reported side effects [31].

### Strengths and limitations

The descriptive design of our study does not allow for statistical comparisons, and our finding of very diverse problems and settings indicate that generalizations should be done carefully. Additionally, although we used direct observation of clinical practice instead of self-reporting to minimize reporting bias, it is possible that health professionals may have performed better than their standard due to the presence of research staff in the consultations [32]. In a study from Tanzania, a significant Hawthorne effect was observed under circumstances comparable to our study, but the effect waned after 10 to 15 consultations [23]. If this finding is generalizable, it has implications for the interpretation of our data, as we observed most health professionals less than 10 to 15 times.

Furthermore, data were collected by a diverse range of researchers with varying background, including medical doctors and research assistants. There is a risk of misclassification when assigning complex acts of clinical consultations into categories on data collection forms and a thorough knowledge of clinical practice is probably an advantage. It is also likely that educational, cultural, and other differences have affected data collection to some extent. However, each country was visited by representatives of the coordinating team (JK, MSØ, AP, and/or SR) prior to study initiation and all forms were thoroughly discussed to minimize reporting differences.

We used a convenience sample of clinical sites, which may have introduced unconsidered biases in our efforts to represent how care is delivered in the participating countries. We also focused on public health services, however, there is a possibility that caregivers frequently bypass the public sector. As such, we may have not identified areas of possible improvement in the management of children with respiratory disease.

### Implications for education, research, health services and policy

Reducing child morbidity and mortality is a core element of the Millennium Development Goals, however, it seems that there is still much ground for improvement in the spectrum of childhood respiratory care particularly in low- and middle-income settings. Increasing awareness and education of health professionals and ensuring availability of essential medications should be among the first steps to combat respiratory misdiagnoses and inappropriate use of antibiotics.

Additionally, economic resources and health system structures vary between countries so attention should be focused on the particularities of each setting. In Kyrgyzstan and Uganda, primary care is mostly manned by mid-level providers, as opposed to Greece and Vietnam where only medical doctors diagnose and treat children. This might partially explain the differences in diagnostic patterns and history taking. In countries with high incidence of pneumonia and limited training of primary care workforce, a focus on adhering to IMCI or other guidelines that are sensitive to pneumonia but not very specific [11] may be an option. However, since most pneumonia and asthma deaths occur in these countries, [2, 12] it seems crucial to develop tools and simple decision support approaches to help health professionals distinguish the most common causes of acute lower respiratory illnesses and refine the differential respiratory diagnosis in young children by studying local etiology of ARI.

Also, over-burdened yet under-staffed facilities may serve to explain the short time of respiratory consultations observed in Uganda and Vietnam. In such circumstances, important aspects of clinical practice may be omitted in a bid to ‘clear’ patient queues. In Uganda, the chest was often not exposed during clinical examination, a finding similar to that of a study from Tanzania [24], leaving health professionals at risk of missing important markers of severe disease. Increasing the focus on enhancing healthcare workforce and strengthening health systems in accordance with the Sustainable Development Goals [33] should be a priority that could facilitate the conduction of consultations with proper history taking and clinical examination [5], factors that are critical for diagnosing respiratory illnesses in children.

The findings of this descriptive study add evidence to the worrying perspective that the quality of clinical assessment for children with respiratory symptoms does not seem to have improved much over the past decade. The deficiencies observed and the differences documented among countries may provide ground for further research to understand the role of potential underlying contextual factors such as culture and quality of doctor-patient relationship, validate trends and inform future interventions.

## Conclusion

There seems to be substantial space for improvement in the management of children under five years of age with respiratory disease in primary care of low-and- middle-, as well as high-income countries. Our study has documented evidence of antibiotic overuse and frequent symptom relief from short-acting β-agonist trials. In countries where diagnosis and treatment of under-fives is done by medical doctors, a focus on increasing education in good antibiotic stewardship and raising awareness regarding differential respiratory diagnosis (including asthma) seems to be crucial. In low-resource settings relying on guidelines to assist primary care providers, a focus on supporting them in performing appropriate history taking and clinical examination by making available delivery devices, clinical decision support applications and essential medications, may constitute important future actions. Strengthening health systems and re-enforcing human resources also seem imperatively needed.

## Supporting information

S1 File Questionnaires in original languages. (PDF)
