# Correction: Diagnosis and treatment of acute respiratory illness in children under five in primary care in low-, middle-, and high-income countries: A descriptive FRESH AIR study

**DOI:** 10.1371/journal.pone.0229680

**Published:** 2020-02-20

**Authors:** Jesper Kjærgaard, Marilena Anastasaki, Marianne Stubbe Østergaard, Elvira Isaeva, Azamat Akylbekov, Nhat Quynh Nguyen, Susanne Reventlow, Christos Lionis, Talant Sooronbaev, Le An Pham, Rebecca Nantanda, James W Stout, Anja Poulsen, FRESH AIR Collaborators

The third author's initials appear incorrectly in the citation. The correct citation is: Kjærgaard J, Anastasaki M, Østergaard MS, Isaeva E, Akylbekov A, Nguyen NQ, et al. (2019) Diagnosis and treatment of acute respiratory illness in children under five in primary care in low-, middle-, and high-income countries: A descriptive FRESH AIR study. PLoS ONE 14(11): e0221389. https://doi.org/10.1371/journal.pone.0221389
