# Supplementary material for: Diagnosis and treatment of acute respiratory illness in children under five in primary care in low-, middle-, and high-income countries: A descriptive FRESH AIR study
Source: PLoS One. 2019 Nov 6;14(11):e0221389. doi: 10.1371/journal.pone.0221389 (PMC6834279; doi:10.1371/journal.pone.0221389)
Supplement: S1 File — (PDF) [file pone.0221389.s001.pdf]

Form 1 - Demographics

Health Worker Observation Checklist

Date

Surveyor ______________________________

Sex ◯ Male ◯ Female

Address (Village) ______________________________

Address (Parish)

Address (Sub-county)

Address (District) ______________________________

~~Cadre of Health worker health worker being observed:~~
◯ ~~Paediatrician~~ ◯ ~~General Doctor~~
◯ ~~Clinical Officer~~ ◯ ~~Degree Nurse~~
◯ ~~Registered Nurse~~ ◯ ~~Enrolled Nurse~~
◯ ~~Registered Midwife~~ ◯ ~~Enrolled Midwife~~
◯ ~~Feldscer~~ ◯ ~~Other~~

~~Specify~~ ______________________________

Cadre of Health worker health worker being observed:
◯ Specialized Paediatrician
◯ Specialized General Doctor
◯ Resident Paediatrician
◯ Resident General Doctor
◯ Degree Nurse
◯ Other

Specify ______________________________

Health worker identifier ______________________________

Time consultation starts (hh:mm) ______________________________

Time consultation ends (hh:mm) ______________________________

Form 1 - Observation of consultation

Health Worker Observation Checklist

1. Presenting complaint a — ◯ Yes ◯ No

~~1. Presenting complaint a (response from caretaker)~~ — ◯ ~~Cough~~ ◯ ~~Difficult breathing~~ ◯ ~~Wheezing~~ ◯ ~~Noisy breathing~~ ◯ ~~Fast breathing~~ ◯ ~~Itching chest~~ ◯ ~~Fever~~ ◯ ~~Other~~

~~Specify~~ ______________________________

1. Presenting complaint a (response from caretaker) — ◯ Cough ◯ Difficult breathing ◯ Wheezing (whistling) ◯ Fast breathing ◯ Dyspnea (kroupsimo) ◯ Fever ◯ Other

Specify ______________________________

2. Presenting complaint b — ◯ Yes ◯ No

~~2. Presenting complaint b (response from caretaker)~~ — ◯ ~~Cough~~ ◯ ~~Difficult breathing~~ ◯ ~~Wheezing~~ ◯ ~~Noisy breathing~~ ◯ ~~Fast breathing~~ ◯ ~~Itching chest~~ ◯ ~~Fever~~ ◯ ~~Other~~

~~Specify~~ ______________________________

2. Presenting complaint b (response from caretaker) — ◯ Cough ◯ Difficult breathing ◯ Wheezing (whistling) ◯ Fast breathing ◯ Dyspnea (kroupsimo) ◯ Fever ◯ Other

Specify ______________________________

3. Presenting complaint c — ◯ Yes ◯ No

~~3. Presenting complaint c (response from caretaker)~~ — ◯ ~~Cough~~ ◯ ~~Difficult breathing~~ ◯ ~~Wheezing~~ ◯ ~~Noisy breathing~~ ◯ ~~Fast breathing~~ ◯ ~~Itching chest~~ ◯ ~~Fever~~ ◯ ~~Other~~

~~Specify~~ ______________________________

3. Presenting complaint c (response from caretaker) — ◯ Cough ◯ Difficult breathing ◯ Wheezing (whistling) ◯ Fast breathing ◯ Dyspnea (kroupsimo) ◯ Fever ◯ Other

Specify ______________________________

4. Other complaints asked — ◯ Yes ◯ No

4. Other complaints asked (response from caretaker) ______________________________

5. Number of days ill asked — ◯ Yes ◯ No

5. Number of days ill asked (response from caretaker) ______________________________

6. Has the health worker asked about the following (if not part of the presenting complaints)?

6a. Duration of cough	◯ Yes ◯ No
6a. Duration of cough (response from caretaker)	______________________________
6b. Night or early morning cough	◯ Yes ◯ No
6b. Night or early morning cough (response from caretaker)	◯ Yes ◯ No
6c. Recurrent cough	◯ Yes ◯ No
6c. Recurrent cough (response from caretaker)	◯ Yes ◯ No
6d. Difficulty in breathing during this illness	◯ Yes ◯ No
6d. Difficulty in breathing during this illness (response from caretaker)	◯ Yes ◯ No
6e. Recurrent difficulty in breathing	◯ Yes ◯ No
6e. Recurrent difficulty in breathing (response from caretaker)	◯ Yes ◯ No
~~6f. Noisy breathing (If YES, note the caretakers description of the breathing)~~	◯ ~~Yes~~ ◯ ~~No~~
6f. Noisy breathing (katsoulia)	◯ Yes ◯ No
~~6f. Noisy breathing (If YES, note the caretakers description of the breathing) (response from caretaker)~~	◯ ~~Yes~~ ◯ ~~No~~
6f. Noisy breathing (response from caretaker)	◯ Yes ◯ No
~~6g.Wheezing during this illness~~	◯ ~~Yes~~ ◯ ~~No~~
6g.Wheezing (whistling) during this illness	◯ Yes ◯ No
~~6g. Wheezing during this illness (response from caretaker)~~	◯ ~~Yes~~ ◯ ~~No~~
6g. Wheezing (whistling) during this illness (response from caretaker)	◯ Yes ◯ N
Triggers asked (Ρωτήθηκαν οι εκλυτικοί παράγοντες)?	◯ Yes ◯ N

7. The above symptoms occur or worsen in presence of (note that questions may only appear following answers to previous questions)

7a. Dusty environment	◯ Yes ◯ No
7a. Dusty environment (response from caretaker)	◯ Yes ◯ No
7b. Respiratory viral infections-flu	◯ Yes ◯ No
7b. Respiratory viral infections-flu (response from caretaker)	◯ Yes ◯ No

~~7c. Biomass smoke~~ ◯ ~~Yes~~ ◯ ~~No~~

7c. Smoke from wood burning ◯ Yes ◯ No

7c. ~~Biomass smoke (response from caretaker)~~ ◯ ~~Yes~~ ◯ ~~No~~

7c. Smoke from wood burning (response from caretaker) ◯ Yes ◯ No

7d. Cigarette smoke ◯ Yes ◯ No

7d. Cigarette smoke (response from caretaker) ◯ Yes ◯ No

~~7e. Aerosol chemicals/sprays~~ ◯ ~~Yes~~ ◯ ~~No~~

7e. Aerosol chemicals/sprays/insecticides ◯ Yes ◯ No

~~7e. Aerosol chemicals/sprays (response from caretaker~~) ◯ ~~Ye~~ ◯ ~~No~~

7e. Aerosol chemicals/sprays/insecticides (response from caretaker) ◯ Yes ◯ No

7f. Changes in temperature (especially cold) ◯ Yes ◯ No

7f. Changes in temperature (especially cold) (response from caretaker) ◯ Yes ◯ No

7g. Animals kept at home (dogs, cats) ◯ Yes ◯ No

7g. Animals kept at home (dogs, cats) (response from caretaker) ◯ Yes ◯ No

7h. During or after exercise ◯ Yes ◯ No

7h. During or after exercise (response from caretaker) ◯ Yes ◯ No

7i. Other triggers noted by caretaker ◯ Yes ◯ No

7i. Other triggers noted by caretaker (response from caretaker) ◯ Yes ◯ No ◯ Other/free text

Specify ________________

History of allergy asked? ◯ Yes ◯ No

---

8. Does the health worker ask about the following? (note that the questions will only appear if the answer to the previous question is 'Yes')

8a. History of allergy in the child ◯ Yes ◯ No

8a. History of allergy in the child (response from caretaker) ◯ Yes ◯ No ◯ Other/free text

Specify ______________________________

8b. Family history of asthma ◯ Yes ◯ No

8b. Family history of asthma (response from caretaker) ◯ Yes ◯ No ◯ Other/free text

Specify ______________________________

8c. Family history of allergy ◯ Yes ◯ No

8c. Family history of allergy (response from caretaker) ◯ Yes ◯ No ◯ Other/free text

Specify ______________________________

Previous medications asked? ◯ Yes ◯ No

Previous medications

8d. i. Salbutamol (oral, nebulized, inhaled) ◯ Yes ◯ No

8d i. Salbutamol (oral, nebulized, inhaled)(response from caretaker) ◯ Oral ◯ Nebulized ◯ Inhaled ◯ No salbutamol ◯ Other

8d. ii. Corticosteroids (oral, injectable, inhaled) ◯ Yes ◯ No

~~8d ii. Corticosteroids (oral, injectable, inhaled)(response from caretaker)~~ ~~Oral ◯ Injectable ◯ Inhaled ◯ No steroids Other~~ ◯ ◯

8d ii. Corticosteroids (oral, injectable, inhaled)(response from caretaker) ◯ Oral ◯ Injectable ◯ Inhaled ◯ No steroids ◯ Other ◯ Mondelukast ◯ Berovent

8d. iii. Antibiotics ◯ Yes ◯ No

8d iii. Antibiotics (response from caretaker) ______________________________

8d. iv. Any other cough medicines ◯ Yes ◯ No

8d iv. Any other cough medicines (response from caretaker) ______________________________

8d. v. Herbal cough medicines ◯ Yes ◯ No

8d v. Herbal cough medicines (response from caretaker) ______________________________

Previous visits to healthcare provider asked? ◯ Yes ◯ No

8e. Previous visits to a healthcare provider (public, private, drug shop) for the same/similar illness

8e. i. Number of visits ◯ Yes ◯ No

8e. i. Number of visits (response from caretaker) ______________________________

8e. ii. Medication given ◯ Yes ◯ No

8e. ii. Medication given (response from caretaker) ______________________________

9. Fever

9a. Fever ◯ Yes ◯ No

9a. Fever (response from caretaker) ____________________

9b. Duration fever ◯ Yes ◯ No

9b. Duration of fever (response from caretaker) ____________________

Form 1 - Clinical signs

10. Weight measured ◯ Yes ◯ No

10. Weight (in kgs, comment) ____________________

11. General condition: Alert, reacts to Verbal, Pain, or Unresponsive ◯ Yes ◯ No

11. General condition: Alert, reacts to Verbal, Pain, or Unresponsive (comment) ◯ Alert ◯ Verbal Pain ◯ Unresponsive

12. Audible wheeze ◯ Yes ◯ No

13. Cyanosis ◯ Yes ◯ No

13. Cyanosis (comment) ◯ Yes ◯ No

14. Temperature (felt/measured) ◯ Yes ◯ No

14. Temperature (felt/measured in degrees celsius) (comment) ____________________

15. Oxygen saturation (SPO2) ◯ Yes ◯ No

15. Oxygen saturation (SPO2 in %) (comment) ____________________

Respiratory system

17. Expose the chest ◯ Yes ◯ No

18. Count the respiratory rate (RR) ◯ Yes ◯ No

Does he count for 1 minute? ◯ Yes ◯ No

18. Count the respiratory rate (RR/breaths per minute) (comment) ____________________

19. Look for lower chest in-drawing (εισολκή θώρακος) ◯ Yes ◯ No

19. Lower chest in-drawing present ◯ Yes ◯ No

20. Stethoscope used? ◯ Yes ◯ No

20a. Sthethoscopic findings

- ☐ Normal
- ☐ Crepitations (τρίζοντες)
- ☐ Crackles (υποτρίζοντες)
- ☐ Wheeze (συριγμός)
- ☐ Rales (ρεγχάζοντες)
- ☐ Ronchi (ρόγχοι)
- ☐ Bronchial respiration (βρογχική αναπνοή)
- ☐ Silent Chest (Πνευμονική σιγή)
- ☐ Other

Specify ____________________

21a. Cardiovascular system

21b. Heart rate/pulse rate counted — ◯ Yes ◯ No

21b. Heart rate/pulse rate counted (bpm) (comment) ________________________________

22. Does the health worker check for the following co-morbidities?

- ☐ Enlarged tonsils
- ☐ Hyperemic pharynx
- ☐ Enlarged inferior turbinates (κάτω ρινικές κογχες)
- ☐ Conjunctivitis (επιπεφυκίτιδα)
- ☐ Eczema/allergic dernatitis

Form 1 - Communication with the caretaker

Does the health worker communicate to the caretaker about the following:

23a. Clinical findings ◯ Yes ◯ No

23a. Clinical findings (comment) ______________________

23b. Possible diagnosis ◯ Yes ◯ No

~~23b. Possible diagnosis (comment)~~
- ☐ ~~Viral infection~~
- ☐ ~~Bronchitis~~
- ☐ ~~Pneumonia~~
- ☐ ~~Bronchiolitis~~
- ☐ ~~Asthma~~
- ☐ ~~Pharyngitis~~
- ☐ ~~Pertussis~~
- ☐ ~~Tuberculosis~~
- ☐ ~~Other~~

23b. Possible diagnosis (comment)
- ☐ Viral infection
- ☐ Bronchitis
- ☐ Pneumonia
- ☐ Bronchiolitis
- ☐ Asthma
- ☐ Pharyngitis
- ☐ Pertussis -κοκκύτη
- ☐ Respiratory infection
- ☐ Tonsilitis
- ☐ Other

~~Specify~~ ______________________

Specify ______________________

23c. Possible causes of the illness ◯ Yes ◯ No

23c. Possible causes of the illness (comment) ______________________

23d. Treatment given/needed ◯ Yes ◯ No

23d. Treatment given/needed (comment) ______________________

23e. Dosing including duration ◯ Yes ◯ No

23e. Dosing including duration (comment) ______________________

23f. Use (mechanism of action) ◯ Yes ◯ No

23f. Use (mechanism of action) (comment) ______________________

23g. Possible side effects ◯ Yes ◯ No

23g. Possible side effects (comment) ______________________

23h. Importance of compliance ◯ Yes ◯ No

23h. Importance of compliance (comment) ______________________

23i. When to return ◯ Yes ◯ No

23i. When to return (comment) ______________________________

---

24. For the children with wheezing, does the health worker communicate the following to the caretaker?

24a. Triggers (εκλυτικοί παράγοντες) ◯ Yes ◯ No

24a. Triggers (comment) ______________________________

24b. The importance of avoiding the triggers ◯ Yes ◯ No

24b. The importance of avoiding the triggers (comment) ______________________________

24c. What to do in case of an attack of wheezing ◯ Yes ◯ No

24c. What to do in case of an attack of wheezing (comment) ______________________________

~~24d. Use of rescue medicines ◯ Yes ◯ No~~

24d. Use of rescue medicines (salbutamol) ◯ Yes ◯ No

~~24d. Use of rescue medicines (comment)~~ ______________________________

24d. Use of rescue medicines (salbutamol)(comment) ______________________________

24e. How to use the rescue medicines(inhaler-where available)-dosing ◯ Yes ◯ No

24e. How to use the rescue medicines(inhaler-where available)-dosing (comment) ______________________________

24f. When to go to health care provider ◯ Yes ◯ No

24f. When to go to health care provider (comment) ______________________________

24g. Does the health worker initiate referral for further assessment ◯ Yes ◯ No

24g. Does the health worker initiate referral for further assessment (comment) ______________________________

25. Does the health worker consult any guidelines? ◯ Yes ◯ No

List the guidelines used ______________________________

25. Does the health worker consult any guidelines? (comment) ______________________________

Comments

____

Form 1 - Data extraction from the patient clinical notes (Greece)

Depending on the country for which you are entering data, questions may not appear.

Temperature measured ◯ Yes ◯ No

Temperature (record) ____________________

Respiratory rate measured ◯ Yes ◯ No

Respiratory rate (record) ____________________

Auscultatory findings recorded? ◯ Yes ◯ No

Ausculatory findings (comment) ____________________

Diagnosis
- ☐ Viral infection
- ☐ Bronchitis
- ☐ Pneumonia
- ☐ Bronchiolitis
- ☐ Asthma
- ☐ Pharyngitis
- ☐ Pertussis-κοκκύτης
- ☐ Respiratory infection
- ☐ Tonsilitis
- ☐ None
- ☐ Other

Specify ____________________

Treatment given (record)
- ☐ Antibiotic
- ☐ Mycolytic (βλενολυτικό)
- ☐ Bronchodilator
- ☐ Oral steroid
- ☐ Inhaled steroid
- ☐ Injection steroid
- ☐ Antiviral drug
- ☐ None
- ☐ Other

Other ____________________

If cough or difficulty in breathing, trial of bronchodilator prescribed and given (record) ◯ Yes ◯ No

If cough or difficulty in breathing, trial of bronchodilator prescribed and given (comment) ____________________

If bronchodilator trial, effect of bronchodilator (record) ◯ Yes ◯ No

If bronchodilator trial, effect of bronchodilator (comment) ____________________

Inhaled corticosteroid-ICS (name) (record) ____________________

Oral corticosteroid (name) (record) ____________________

Injectable corticosteroid (name) (record) ______________________________

Antibiotics (name) (record) ______________________________

Oxygen supplementation (record) ◯ Yes ◯ No

Other treatment (name) (record) ______________________________

Other treatment (name) (comment) ______________________________

Sent for investigation? ◯ Yes ◯ No

Sent for investigation
- ☐ X-ray
- ☐ Blood analysis
- ☐ Urine analysis
- ☐ Other

Specify ______________________________

Sent for investigation (comment) ______________________________

Referred or home treatment (record) ◯ R ◯ H

Referred or home treatment (comment) ______________________________

Follow up arranged (record) ______________________________

Follow up arranged (comment) ______________________________

Comments

____

Form 2 - Caretaker interview

Date: ______________________________

Research assistant: ______________________________

Telephone number of caretaker: ______________________________

Patient ID ______________________

---

1. Did the health worker talk to you about the following? (σας μίλησε ο γιατρός για τα)

1a. Clinical findings (κλινικά ευρήματα) ◯ Yes ◯ No

1a. Clinical findings (comment) ______________________________

1b. Possible causes of the disease (πιθανές αιτίες) ◯ Yes ◯ No

1b. Possible causes of the disease (comment) ______________________________

1c.The medicine he/she prescribed (φάρμακο που συνταγογραφ) ◯ Yes ◯ No

1c. The medicine he/she prescribed (comment) ______________________________

1d. How the medicine works (πώς λειτουργεί το φάρμακο) ◯ Yes ◯ No

1d. How the medicine works (comment) ______________________________

1e. Dosing/frequency (Δοσολογία/συχνότητα) ◯ Yes ◯ No

1e. Dosing/frequency (comment) ______________________________

1f. Duration of treatment (διάρκεια θεραπείας) ◯ Yes ◯ No

1f. Duration of treatment (comment) ______________________________

1g. When to return immediately (πότε να επιστρέψετε άμεσα) ◯ Yes ◯ No

1g. When to return immediately (comment) ______________________________

1h. For follow up (για επανεξέταση) ◯ Yes ◯ No

1h. For follow up (comment) ______________________________

For children with wheezing, did the health worker talk about: (για τα παιδιά με συριγμό)

2a. Triggers (εκλυτικοί παράγοντες) ◯ Yes ◯ No

2a. Triggers (comment) ______________________________

2b. What to do in case of another attack of wheezing ◯ Yes ◯ No

2b. What to do in case of another attack of wheezing (comment) ______________________________

2c. The medicine you should give to your child if he/she starts wheezing ◯ Yes ◯ No

2c. The medicine you should give to your child if he/she starts wheezing (comment) ______________________________

2d. How to administer it (πώς να το χορηγήσετε) ◯ ◯
Yes No

2d. How to administer it (comment) ______________________________

Form 2 - Part II

3a. Household smoking? ◯ Yes ◯ No

4a. Has your child been exposed to biomass smoke? ◯ Yes ◯ No

4b. How many months of his/her life has he/she been exposed? (comment) ______________________________

4c. On average how many hours /day is he/she exposed? (comment) ______________________________

4d. Were you (the mother) of the child exposed in pregnancy? ◯ Yes ◯ No

---

Socio-demographic indicators:

5. Number of years of maternal education ______________________________

5a. Maternal age (in years) ______________________________

5b. Is the child a twin? ◯ Yes ◯ No

5c. Number of siblings ______________________________

6a. Previous attendance for cough/difficult breathing? ◯ Yes ◯ No

6b. Number of previous attendances for cough/difficult breathing (comment) ______________________________

6c. Cough for more than 14 days? ◯ Yes ◯ No

7. Last vaccine given
◯ BCG ◯ Polio 0 ◯ DPT-Hep B +Hib -1+ Polio 1 ◯ DPT-Hep B +Hib -2+ Polio 2
◯ DPT-Hep B +Hib -3+ Polio 3
◯ PCV 1 ◯ PCV 2 ◯ PCV 3
◯ Measles

BCG: φυματίωση

Polio: πολιομυελίτιδα

DPT: διφθερίτιδα

Hep B : Ηπατίτιδα Β

Hib: Αιμόφιλου Γρίπης

PCV: Πνευμονιόκοκκου

Measles: ιλαρά

Form 3 - Follow up

Surveyor ______________________

Date: ______________________

Patient ID ____________________________________

Follow-up 5 days

	Yes	No
Contact achieved		
Fever	◯	◯
Still difficult breathing	◯	◯
Attended scheduled follow-up at HC	◯	◯
	◯	◯
Admitted to hospital Child	◯	◯
alive	◯	◯
Compliance to medication	◯	◯

1. Form 4 - Introduction.

Record ID ______________________

1. Country
   - ◯ Greece
   - ◯ Kyrgyzstan
   - ◯ Uganda
   - ◯ Vietnam

2. Health facility No. ______________________

3a. Title of post: ______________________
(first provider)

3a. Date ______________________
(first provider)

3b. Title of post: ______________________
(second provider)

3b. Date ______________________
(second provider)

IMCI within the health facility:

4. Do you have an IMCI focal person within the health facility? ◯ Yes ◯ No ◯ Don't know

5. How many staff members have been trained in the IMCI strategy? ______________________

6. And how many of these within 2015? ______________________

7. Any other kind of training for paediatric staff has taken place in 2015:

1: ______________________

2: ______________________

3: ______________________

4: ______________________

8. Has the health facility received economic/material support from other sources than the public?

8a. From church organisation ◯ Yes ◯ No

List: ______________________

8b. Private NGO's ◯ Yes ◯ No

List: ______________________

8c. Other organisations ◯ Yes ◯ No

List: __________________________________

2. Form 4 - General information

9. Number of beds in the paediatric department/ward: ______________________

10. Number of cots for neonatal/young infant admissions: ______________________

11. Annual number of deliveries at the hospital: ______________________

Paediatricians

12a. Annual number of paediatricians: ______________________
(paediatric ward)

12b. Number of unfilled paediatrician posts: ______________________

Doctors

12c. Annual number of doctors: ______________________
(health facility)

12d. Annual number of doctors: ______________________
(paediatric ward)

12e. Number of unfilled doctors posts: ______________________

Medical assistants

12f. Annual number of medical assistants: ______________________
(health facility)

12g. Annual number of medical assistants: ______________________
(paediatric ward)

12h. Number of unfilled medical assistant posts: ______________________

Nurses

12i. Annual number of nurses: ______________________
(health facility (dayshift))

12j. Annual number of nurses: ______________________
(health facility (nightshift))

12k. Annual number of nurses: ______________________
(paediatric ward (dayshift))

12l. Annual number of nurses: ______________________
(paediatric ward (nightshift))

12m. Number of unfilled nurses posts: ______________________
(dayshift)

12n. Number of unfilled nurses posts: ______________________ (nightshift)

Auxiliary staff

12o. Annual number of auxiliary staff: ______________________ (health facility (dayshift))

12p. Annual number of auxiliary staff: ______________________ (health facility (nightshift))

12q. Annual number of auxiliary staff: ______________________ (paediatric ward (dayshift))

12r. Annual number of auxiliary staff: ______________________ (paediatric ward (nightshift))

12s. Number of unfilled auxiliary staff posts: ______________________ (dayshift)

12t. Number of unfilled auxiliary staff posts: ______________________ (nightshift)

3. Form 4 - Triage and treatment (ETAT) for children waiting to be seen

13. Is there any form of triage of patients attending the health facility? ◯ Yes ◯ No

If yes: ______________________________

14. Who is responsible for triage? ______________________________

15. Are there any guidelines on triage in the health facility? ◯ Yes ◯ No

Please describe: ______________________________

16. Describe the triage categories and what happens to each category: ______________________________

4. Form 4 - Services And Facilities Available

---

17. Services and facilities available

	Always	Mostly	Rarely	Never
Do you have electricity at your health facility?	○	○	○	○
Do you have running water?	○	○	○	○
Do you have soap and /or disinfectant for hand-washing in the paediatric ward?	○	○	○	○
Are the patient washing and toilet facilities adequate?	○	○	○	○
Do children share beds with one-another?	○	○	○	○
Is transport available to send referrals?	○	○	○	○

5. Form 4 - Available Equipment

18. Is the following equipment available in your hospital:

	Always	Often	Rarely	Never
Oxygen?	○	○	○	○
Flow-meters for oxygen?	○	○	○	○
Oxygen saturation monitor?	○	○	○	○
Equipment for the administration of oxygen?	○	○	○	○
IV-giving sets with chambers for paediatric use? (burette)	○	○	○	○
Butterflies and/or cannulas of paediatric size?	○	○	○	○
NG-tubes, paediatric size?	○	○	○	○
Equipment for intra-osseous fluid administration?	○	○	○	○
Suction equipment?	○	○	○	○
Nebulizers for administration of salbutamol?	○	○	○	○
Spacers with masks for administration of metered doses (spray) of salbutamol?	○	○	○	○
Functional X-ray equipment?	○	○	○	○
Stethoscopes?	○	○	○	○
Thermometers?	○	○	○	○

	Always	Often	Rarely	Never	NA
Routines for cleaning inhalation equipment in place?	○	○	○	○	○

Indicate source:
- ○ Oxygen cylinder
- ○ Oxygen concentrator
- ○ Central supply

6. Form 4 - Observational check list: all inspection

19. Are there written guidelines available for assessing the child with breathing difficulty/Acute respiratory illness? ○ Yes ○ No

20. Which are available?
☐ IMCI Wallchart
☐ Local guidelines
☐ IMCI flipchart
☐ Other

Describe: ____________________

21. Look for and record presence of the following on the health facility:

	Yes	No	NA
Ambu-bag and masks of different sizes?	○	○	○
Scales for newborns?	○	○	○
Scales for children?	○	○	○
Nebulizers?	○	○	○
Spacers for inhalation medicine metered dose administration?	○	○	○
Are there guidelines for cleaning nebulizers?	○	○	○
Does inhalation equipment look clean?	○	○	○
Thermometers?	○	○	○
Stethoscopes?	○	○	○

22. Antiasthmatic medicine (from WHO essential drugs list):

	Yes	No
Budesonide, Inhalation (aerosol): 1. 100 micrograms per dose;	○	○
2. 200 micrograms per dose;	○	○
Salbutamol: 1. Injection, 50 micrograms (as sulfate)/mL in 5-mL ampoule.	○	○
2. Metered dose inhaler (aerosol): 100 micrograms (as sulfate) per dose.	○	○
3. Respirator solution for use in nebulizers: 5 mg (as sulfate)/mL.	○	○
4. Oral Salbutamol (not on essential drugs list)	○	○
Other inhaled cortico-steroid:	○	○
Other short acting beta-2 agonist:	○	○
Oral cortico-steroid:	○	○

Other inhaled cortico-steroid: ______________________________

Other short acting beta-2 agonist: ______________________________

Oral cortico-steroid: ______________________________

7. Form 5 - Introduction

Number referral sites __________________________________

Information provided by (title of post) __________________________________

Information provided by (title of post) __________________________________

8. Form 5 - General information

Use N/A to indicate questions that are not applicable.

1. Number of beds in the paediatric department/ward: ______________________

2. Number of cots for neonatal/young infant admissions: ______________________

3. Annual number of deliveries at the hospital: ______________________

Paediatricians

4a. Average number of paediatricians: ______________________ (paediatric ward)

4b. Number of unfilled paediatric posts: ______________________

Doctors

4c. Average number of doctors: ______________________ (hospital)

4d. Average number of doctors: ______________________ (paediatric ward)

4e. Number of unfilled doctor posts: ______________________

Medical assistants

4f. Average number of medical assistants: ______________________ (hospital)

4g. Average number of medical assistants ______________________ (paediatric ward)

4h. Number of unfilled medical assistant posts: ______________________

Nurses

4i. Average number of nurses: ______________________ (hospital (dayshift))

4j. Average number of nurses: ______________________ (hospital (nightshift))

4k. Average number of nurses: ______________________ (paediatric ward (dayshift))

4l. Average number of nurses: ______________________ (paediatric ward (nightshift))

4m. Number of unfilled nurse posts: ______________________ (dayshift)

4n. Number of unfilled nurse posts: ______________________ (nightshift)

Auxiliary staff

4o. Average number of auxiliary staff: ______________________ (hospital (dayshift))

4p. Average number of auxiliary staff: ______________________ (hospital (nightshift))

4q. Average number of auxiliary staff: ______________________ (paediatric ward (dayshift))

4r. Average number of auxiliary staff: ______________________ (paediatric ward (nightshift))

4s. Number of unfilled auxiliary staff posts: ______________________ (dayshift)

4t. Number of unfilled auxiliary staff posts: ______________________ (nightshift)

5. Services and facilities available

	Always	Mostly	Rarely	Never
Do you have electricity at your health facility?	○	○	○	○
Do you have running water?	○	○	○	○
Do you have soap and/or disinfectant for hand-washing in the paediatric ward?	○	○	○	○
Are the patient washing and toilet facilities adequate?	○	○	○	○
Do children share beds with one-another?	○	○	○	○
Is transport available to send referrals?	○	○	○	○

6. Is the following equipment available in your hospital?

	Always	Often	Rarely	Never
Oxygen?	○	○	○	○
Flow-meters for oxygen?	○	○	○	○
Oxygen saturation monitor?	○	○	○	○
Equipment for the administration of oxygen?	○	○	○	○
IV-giving sets with chambers for paediatric use? (burette)	○	○	○	○
Butterflies and/or cannulas of paediatric size?	○	○	○	○
NG-tubes, pediatric size?	○	○	○	○
Equipment for intra-osseous fluid administration?	○	○	○	○
Suction equipment?	○	○	○	○
Nebulisers for administration of salbutamol?	○	○	○	○
Spacers with masks for administration of metered doses (spray of salbutamol)?	○	○	○	○
Functional X-ray equipment?	○	○	○	○

Indicate oxygen source:

- ☐ Oxygen cylinder
- ☐ Oxygen concentrator
- ☐ Central supply

9. Form 5 - Observational Checklist (All Inspection)

7a. Breathing difficulty/Acute respiratory illness

Look for and record presence of the following at the ward:

	Yes	No
Ambu-bag and masks of different sizes?	◯	◯
Scales for newborns?	◯	◯
Scales for children?	◯	◯
Nebulizers?	◯	◯
Spacers for inhalation medicine metered dose administration?	◯	◯
Thermometers?	◯	◯
Stethoscopes?	◯	◯

7b. Antiasthmatic medicine (from WHO essential drugs list):

	Yes	No
Budesonide, Inhalation (aerosol):	◯	◯
1.100 micrograms per dose;	◯	◯
2.200 micrograms per dose	◯	◯
Salbutamol:	◯	◯
1.Injection, 50 micrograms (as sulfate)/mL in 5-mL ampoule. 2:	◯	◯
2.Metered dose inhaler (aerosol): 100 micrograms (as sulfate) per dose.	◯	◯
3.Respirator solution for use in nebulizers: 5 mg (as sulfate)/mL.	◯	◯
4.Oral Salbutamol	◯	◯
Other inhaled cortico-steroid:	◯	◯
Other short acting beta-2 agonist:	◯	◯

Name other inhaled cortico-steroid: ________________________________

Name other short acting beta-2 agonist: ________________________________

Form 3 - Follow up

Surveyor ____________________

Date: ____________________

Patient ID ____________________

Follow-up 5 days

	Yes	No
Contact achieved		
Fever	◯	◯
Still difficult breathing	◯	◯
Attended scheduled follow-up at HC	◯	◯
	◯	◯
Admitted to hospital Child	◯	◯
alive	◯	◯
Compliance to medication	◯	◯

FRESH AIR Form 2: Caretaker interview

Enanba y'omulwaire____________________

Enaku edh'omwezi________________________________

Ayamba kwanonenkereza__________________________________

No.	Item	Response		Comment
		Yii	Bbe	
1	Owmusawo ayogeireku niiwe kubino ghansi:			
a	Byazwiire ebirikukuluma			
b	Ebireeta endwaire eyo			
c	Obulezi bwakuwandhikiire			
d	Engeri obulezi yebukolamu			
e	Ekigero ekyobulezi bwolinokumira			
f	Eibanga lyolinokumala kubwidhandhabi			
g	Li lwolinokwira Olinakwiriragho			
h	Kulondoola			
2	Eri abaana abali kugheera nga bakyoolya, omusagho akyogeireku:			
a	Ebikireeta			
b	Kiki ekyokukola singa obanga olumbiibwa okugheera nga okyoolya okundhi			
c	Obulezi obusobola okukutaasa			
d	Engeri yoyonza okubughamu omuntu			

PART II

	Item	Response (Y/N)	Comment
3a	Okufugheeta sigala mumaka?		
b	Okufugheeta sigala bulirunaku abaana ghebaba?		
4a	Omwanawo abaire afuna okunyokerwa omwoosi?		
4b	Ghabanga abaire anyokerwa omwoosi, amaze myezi emeka nga anyokerwa omwoosi?		
4c	Okugerageranhia sawa nga imeka/enaku dhafuna okunyokerwa omwoosi?		
4d	Wafuna (maama womwana) okunyokerwa omwoosi nga alindha?		
5	Ebiraga ebikugemaku:		
5a	Emyaka gya maama		
5b	Omwanawo mulongo		
5c	Baganda ni banhina bomwana bali bameka?		
6a	Eibanga eribise wajaku okufuna obuyambi kubuzibu obwokukolola/obuzibu mukugheera		
6b	Emirundi jewaja eibanga eribise okufuna obuyambi kubuzibu obwokukolola/obuzibu mukugheera		
6c	Okukolola kwaswika munaku 14?		
7	Erina eryobulezi obugema obwakugheebwa		Enaku edh'omwezi

Enamba ey'eisimu…………………………………………………………..

Amaina agobulezi obugema

- BCG Obugema akafuba
- Polio 0 Obugema poliyo
- DPT-Hep B +Hib -1 Doozi esooka egheebwa kumyezi ebiri okutangira endwaire ningi nga mwotwalire tatanansi, poliyo akalakiiro nedhindhi
- DPT-Hep B +Hib-2 Doozi eyokubiri egheebwa kumyezi ena okutangira endwaire ningi nga mwotwalire tatanansi, poliyo akalakiiro nedhindhi
- DPT- Hep B +Hib-3 Doozi eyokusatu egheebwa kumyezi mukaaga okutangira endwaire ningi nga mwotwalire tatanansi, poliyo akalakiiro nedhindhi
- PCV 1 Doozi esooka okutangira endwaire ningi nga mwotwalire mulalama, nedhindhi edhikosa ekifuba

- PCV 2 Doozi eyokubiri okutangira endwaire ningi nga mwotwalire mulalama, nedhindhi edhikosa ekifuba
- PCV 3 Doozi eyokusatu okutangira endwaire ningi nga mwotwalire mulalama, nedhindhi edhikosa ekifuba
- Measles (obwolunkusense)

Приложение A: регистрационная карта
Appendix A: Enrolment card

Регистрационная карта
Enrolment Card

Дата:___/___/___ Название учреждения:________________Код:_________
Date Facility name Code

Время пребывания ребенка в учреждении:_____часы______мин
Child's arrival time at the facility hours min

Идентификационный номер ребенка:________________________________
Child's ID

Имя ребенка:____________________________Дата рождения:____/____/____

Возраст (в месяцах):_________________
Age (months)

Пол ребенка: (1) М (2) Ж
Child sex M F

Прочтите следующее заявление для опекуна.
Read the following statement to the caretaker.

"Здравствуйте. Я _______________ – представитель исследовательской группы Fresh Air. Я здесь со своими коллегами для того, чтобы провести исследование у детей с частыми заболеваниями, а также как они обычно обследуются и лечатся в организациях здравоохранения. Член нашей команды хотел бы понаблюдать за консультацией детей медицинским работником. После этой консультации другой член нашей команды хотел бы задать вам несколько основных вопросов. Нет никаких рисков или прямой выгоды для вас от участия в опросе, но ваше участие будет способствовать улучшению медицинского обслуживания в данной местности и в других. Ваш ребенок будет осмотрен и пролечен, если вы решите не участвовать. Будьте уверены, что информация будет конфиденциальной, и вы можете прервать свое участие в любое время или воздержаться от ответов на любые вопросы".

"Hello. I am _______________ from the Fresh Air research group. I am here with my colleagues to do a study about children with common illnesses, and how they are routinely assessed and treated in health facilities. A member of our team would like to observe the consultation between your child and the health worker. Following your consultation another member of our team would like to ask you some background questions. There are no risks or direct benefits to you from participating in the survey, but your participation will contribute to improving health services in this and other facilities. Your child will still be seen and treated if you do not choose to participate. Please be assured that the information will be confidential and you may end your participation at any time or refrain from answering any questions."

«Есть ли у вас вопросы по поводу этого исследования в данный момент?
"At this time, do you want to ask me anything about this survey?"

«Согласны ли вы принять участие в этом исследовании?»
"Do I have your agreement to participate?"

Подпись:____
Signature

Приложение С: Форма 1 Код наблюдения за консультацией
Appendix C: Form 1 Code Observation of consultation

Базовое исследование FRESH AIR WP6 - Форма 1<br>FRESH AIR WP6 Baseline survey - Form 1<br>Item/Пункт<br>Data/дата<br>Identifier/идентификатор		Опрошено/Проверено<br>Asked/Checked	(Да/Нет) Ответ<br>(Y/N) Answer	Идентификационный<br>Номер пациента<br>Patient idno:<br><br>Формат данных<br>Data formate
**Предпосылки**<br>**Background**				
f100	Местонахождение исследования FRESH AIR<br>FRESH AIR study site			
f101	Обучение медицинского работника<br>Health worker education			
f102	Идентификатор медицинского работника<br>Health worker identifier			
f103	Дата<br>Date			
f104	Начало консультации<br>Start of consult			Час/мин<br>Hour/min
f105	Окончание консультации<br>End of consult			Час/мин<br>Hour/min
f106	Возраст ребенка<br>Child age			Месяцы<br>Months
f107	Пол ребенка<br>Child sex			М/Ж<br>M/F
f108	Исследователь<br>Surveyor			Инициалы<br>Initials

**Наблюдение за консультацией**<br><br>**Observation of consultation**				
f109	Опрошен на наличие жалоб?<br><br>Presenting problems asked?			
f109a	Наличие жалоб a<br><br>Presenting problem a			
f109b	Наличие жалоб b<br><br>Presenting problem b			
f109c	Наличие жалоб c<br><br>Presenting problem c			
f109d	Опрошен ли по поводу других жалоб?<br><br>Other history asked?			
f110	Количество дней заболевания<br><br>Number of days ill			Количество дней<br><br>Number of days
f111	Ребенок: в сознании, реакция на голос, реакция на боль, без сознания.<br><br>Child: **A**lert, reacts to **V**erbal, **P**ain, or **U**nresponsive			С/Г/Б/БС<br><br>A/V/P/U
f112	Опрошен по поводу способности кушать?<br><br>Able to feed asked?			(Да/Нет)<br>(Y/N)
f113	Есть ли рвота?<br><br>Vomiting everything?			(Да/Нет)<br>(Y/N)
f114	Есть ли сейчас судороги?<br><br>Convulsing now?			(Да/Нет)<br>(Y/N)
f115	Летаргия/кома<br><br>Lethargy / coma			(Да/Нет)<br>(Y/N)
f116	Проверен ли на ригидность шеи?<br><br>Stiff neck checked?			(Да/Нет)<br>(Y/N)
f118	Есть ли лихорадка?<br><br>Fever a problem			(Да/Нет)<br>(Y/N)
f119	Как долго есть лихорадка?<br><br>Fever how long asked?			(Да/Нет)<br>(Y/N)
f120	Кашляет?<br><br>Coughing?			(Да/Нет)<br>(Y/N)
f121	Затрудненное дыхание?<br><br>Difficult breathing?			(Да/Нет)<br>(Y/N)

f122	Было ли слышно дома свистящее дыхание/хрипы/хруст?<br><br>Audible wheezing/raling/crackling at home?			(Да/Нет)<br>(Y/N)
f127	Тяжелый кашель/затрудненное дыхание?<br><br>Severe cough/difficult breathing a problem?			(Да/Нет)<br>(Y/N)
f128	Ночной кашель/ затрудненное дыхание?<br><br>Night cough/difficult breathing a problem?			(Да/Нет)<br>(Y/N)
f129	Предыдущие эпизоды таких симптомов?<br><br>Previous episodes with same symptoms?			Количество эпизодов<br><br>Number of episodes
f130	Как долго длится кашель/затрудненное дыхание<br><br>Cough/difficult breathing for how long asked			(Да/Нет)<br>(Y/N)
**Диагноз и лечение**<br><br>**Diagnosis and treatment**				
f132	Диагноз? (спросить после консультации при не упоминании)<br><br>Diagnosis? (Ask after consultation of not mentioned)			Строки<br><br>String
f133	Если есть кашель/затрудненное дыхание, пытались ли использовать бронходилятатор?<br><br>If cough/ difficult breathing, trial of bronchodilator?			
f134	Если пользовался бронходилятатором, был ли эффект от бронходилятатора?<br><br>If bronchodilator trial, effect of bronchodilator?			(Да/Нет)<br>(Y/N)
f135	Было ли назначено лечение?<br><br>Treatment given?			
f136	Ингаляционные кортикостероиды (название)<br><br>ICS (name)			

f137	Оральные кортикостероиды (название)<br><br>Oral corticosteroid (name)			
f138	Бронходилятоторы<br><br>BD (name)			
f139	Антибиотики (название)<br><br>Antibiotics (name)			
f140	Был ли дан дополнительный кислород?<br><br>Supplemental oxygen given?			
f141	Если был дан какой-то ингаляционный препарат (БД, ИКС, 02), то какие приспособления ребенок использовал? обработанные/ необработанные, или новые приспособления?<br><br>If any inhalation treatment (BD? ICS? 02) is given? Is the child supplied with an uncleaned/cleaned or new inhalation device?			НО/О/Н<br><br>U/C/N
f142	Другие препараты (названия)<br><br>Other treatment (name)			
F143	Направлен на исследование (какое?)<br><br>Sent for investigation (which?)			
f144	Следующий уровень или домашнее лечение<br><br>Refer or home treatment			С/Д<br><br>R/H
F146	Следующий визит<br><br>Follow up arranged			Через сколько дней<br><br>How many days later
**Клинические признаки**<br><br>**Clinical signs**				
f147	Вес<br><br>Weight			кг<br><br>kg
f148	Подсчитана ли частота дыхания?<br><br>Respiratory rate counted?			ЧД/мин

				breaths/min
f149	Осмотрена ли грудная клетка<br><br>Chest exposed and observed?			(втяжение Есть/Нет)<br><br>(indrawing Y/N)
f150	Подсчитана ли частота пульса?<br><br>Pulse counted?			ЧСС/мин<br><br>beats/min
f151	Выявлен ли на цианоз?<br><br>Cyanosis checked			(Да/Нет)<br>(Y/N)
f152	Измерена ли температура тела? ощущается?<br><br>Temperature felt/measured?			°C
f153	Измерена ли сатурация?<br><br>Saturation measured?			%
f154	Имеются ли дистантные хрипы?<br><br>Audible wheeze present?			(Да/Нет)<br>(Y/N)
f155	Был ли использован стетоскоп? (если был использован, спросите медработника о результатах после консультации)<br><br>Stethoscope used? (if used, ask provider for result after consultation)			1: в норме (normal), 2: крепитация (crepitations), 3: **хруст (crackles),** 4: одышка (wheeze), 5: хрипы (rales),6: шум (ronchi), 7:бронхиальное дыхание (bronchial respiration), 8: "немое" легкое (silent chest), 9: другое (other)
f156	Имеется ли стридор?<br><br>stridor present			(Да/Нет)<br>(Y/N)
Комментарии:<br>Comments:				

Приложение D: Форма 2 Беседа с родителем
Appendix D: Form 2 Caretaker interview

FRESH AIR Form 2. Clinical signs, caretaker interview and follow-up.
Patient idno:__________
FRESH AIR Форма 2. Клинические признаки, беседа с родителем и последующие мероприятия.
Идентификационный номер пациента:__________

Имя пациента:_____________________Дата:___________________Исследователь:_______________ ____
(patient name) (date) (surveyor)

Беседа с родителем:____ _____
(caretaker interview)

**Вопрос:**<br><br>**Question:**			**Разъяснение:**<br><br>**Explanation:**
CI1	Курение внутри дома (Да/Нет)<br><br>Household smoking (Y/N)		Любое курение вблизи от ребенка<br><br>Any smoking daily at child's residence
CI2	Подвергался ли Ваш ребенок воздействию сжигания биомасс?<br><br>Has your child been exposed to biomass smoke (or whatever term used) (Y/N)?		
CI2a	Если да, то сколько месяцев он/она подвергался (-ась) в течение своей жизни?<br><br>If yes, how many months of his/her life has he/she been exposed?		Месяцы<br><br>Months
CI2b	Сколько часов в день подвергается воздействию?<br><br>On average how many hours/ day is he/ she exposed?		Часы<br><br>Hours
CI3	Подвергалась ли мать ребенка воздействию сжигания биомасс во время беременности? (Да/Нет)<br><br>Were you/the mother of the child exposed in pregnancy (Y/N)		

CI4	Уровень образования матери?<br><br>Socio-demographic indicators		Данные вводит ведущая страна
CI5	Возраст матери<br><br>Maternal age (years)		
CI6	Близнецы (Да/Нет)<br><br>Twin (Y/N)		
CI7	Родные братья и сестры (количество)<br><br>Child siblings (number)		
CI8	Было ли предыдущее обращение по поводу кашля/затрудненного дыхания? (Да/Нет)<br><br>Previous attendance for cough/difficult breathing (Y/N)		
	Number of Previous attendance for cough/difficult breathing<br><br>Количество предыдущих обращений по поводу кашля/затрудненного дыхания?		
CI9	Кашель больше 14 дней? (Да/Нет)<br><br>Cough for more than 14 days? (Y/N)		
CI10	Последняя полученная вакцина (название)Last vaccine given (name)		Дата:<br><br>Date:_________________

Имя: _______________________________________
Номер телефона:___________________________

Названия вакцин:

БЦЖ
АКДС + Hib-1
АКДС + Hib-2
АКДС + Hib-3
Корь 1
Корь 2
ОПВ-0
ОПВ-1
ОПВ-2
ОПВ-3
Гепатит В0
Гепатит В1
Гепатит В2
Гепатит В3
РТВ-1
РТВ-2

ПКВ-1
ПКВ-2
ПКВ-3

Приложение E: Форма 3 Последующие мероприятия
Appendix E: Form 3 Follow-up

FRESH AIR Форма 3. Последующие мероприятия на 5-й день.
FRESH AIR Form 3. Follow-up at day 5.

ID пациента__________ Имя ребенка: _______________ Исследоваель: _______
Patient idno:__________ Child name: _________________ Surveyor: _______

Последующие мероприятия на 5-й день: (Follow-up 5 days)

Контакт достигнут (Да/Нет) (Contact achieved (Y/N)): ___________

Дата (Date): ___________________________

Лихорадка (Да/Нет) (Fever (Y/N)) ___________________

Все еще кашляет (Да/Нет) (Still coughing(Y/N))__________

Все еще затрудненное дыхание (Да/Нет) (Still difficult breathing (Y/N)) __________

Последующая деятельность в медучреждении запланирована
(Да/Нет) (Attended scheduled follow-up at HC (Y/N)) ___________

Госпитализирован (Да/Нет) (Admitted to hospital (Y/N)): ________

Ребенок жив (Да/Нет) (Child alive
(Y/N)): _________________

Приложение F: Форма 3 Беседа с командой администрации медучреждения
Appendix F: Form 3 Interview Guide Health facility management team

Форма 4 Fresh Air: Команда управления медучреждения.

Страна:________________________ Медучреждение №.________________

Form 4 Fresh Air: Health facility management team.

Country:________________________ Health facility No.__________________

ПОЛУЧЕННЫЕ ДАННЫЕ ИЗ БЕСЕДЫ СО СТАРШИМИ ОТВЕТСТВЕННЫМИ СОТРУДНИКАМИ
DATA OBTAINED BY INTERVIEW WITH RESPONSIBLE SENIOR STAFF

**Information Provided by (Title of post)**<br>**Информация предоставлена (наименование должности)**	**Дата**<br>**Date**

ИВБДВ в медицинском учреждении:
IMCI within the health facility:

Есть ли в вашем медицинском учреждении координаторы по ИВБДВ Да/Нет/Не знаю
Do you have a IMCI focal person within the health facility Y/N/don't know
Сколько сотрудников прошли обучение стратегии ИВБДВ:__________________, и сколько из них в течение 2015 года:______________
How many staff members have been trained in the IMCI strategy:_________________, and how many of these within 2015:____________________

Любое другое обучение педиатрического персонала, состоявшееся в 2015 году:
Any other kind of training for paediatric staff has taken place in 2015:

1. ____
2. ____
3. ____
4. ____

Получало ли медицинское учреждение экономическую/ материальную поддержку из источников кроме государственных?

Has the health facility received economic/ material support from other sources than the public?

1. От религиозных организаций Да/Нет Список:__________________________
From church organisation Y/N List
2. Частные НПО Да/Нет Список:__________________________
Private NGO's Y/N List
3. Другие организации Да/Нет Список:__________________________
Other organizations Y/N List

Введение:
Introduction

Цель – описать ресурсы, имеющиеся в учреждении.
The purpose is to describe the resources available to the facility.

Общая информация (если неприменимо, указать на Н/А):

General information (if not applicable, indicate by N/A):

Количество коек в педиатрическом отделении/
Number of beds in the paediatric department/
палата? Количество детских кроваток для новорожденных/младенцев
ward? Number of cots for neonatal / young infant
приемная? Ежегодное число родов в больнице?
admissions?Annual number of deliveries at the hospital?

	Койки (Beds)
	Кроватки (Cots)
	Ежегодно (per year)

Что такое **фактическая** (средняя) численность персонала в больнице и в педиатрической палате (-тах):
What is the **actual** (average) number of staff at the hospital and in the paediatric ward(s):

	Мед учреждение (все отд-я) Health facility (all *********dept.)	Педиатрическое отделение/па-лата (кол-во незаполненных мест)	Paediatric dept/ward (No. of unfilled posts)
Педиатры? (Paediatricians?)			
Семейные врачи? (Doctors? (MOs))			
Мед. ассистенты? (Medical assistants? (COs))			
Медсестры в дневную смену? (Nurses per dayshift? (NDs))			
Медсестры в ночную смену? (Nurses during the nightshift? (NNs))			

Вспомогательный персонал в дневную смену? (Auxiliary staff per dayshift (ADs))		
Вспомогательный персонал в ночную смену? (Auxiliary staff during the nightshift (ANs))		

Сортировка и лечение (ETAT) детей, ожидающих осмотр
Triage and Treatment (ETAT) for children waiting to be seen

Есть ли форма сортировки пациентов, посещающих медицинское учреждение?<br>Is there any form of triage of patients attending the health facility?	Да/Нет<br>Y/N
Если есть:<br>If YES:	
Кто несет ответственность за сортировку?<br>Who is responsible for triage?	
Существуют ли какие-нибудь рекомендации по сортировке в медицинском учреждении? Если это так, то опишите, пожалуйста.<br>Are there any guidelines on triage in the health facility? If so please describe	
Опишите категории сортировки и что происходит с каждой категорией?<br>Describe the triage categories and what happens to each category?	

Доступные условия и удобства (есть=отмечаете, нет=оставляете пустым)
Services and facilities available: (tick=yes, blank=no)

	Всегда<br>Always	Зачастую<br>Mostly	Редко<br>Rarely	Никогда<br>Never
Есть ли электричество в вашем медучреждении?<br>Do you have electricity at your health facility?				
Есть ли проточная вода?<br>Do you have running water?				
У вас есть мыло и/или дезинфицирующее средство для мытья рук в детском отделении?<br>Do you have soap and /or disinfectant for hand-washing in the paediatric ward?				
Являются ли процедуры мытья и туалетные удобства адекватными для пациента?<br>Are the patient washing and toilet facilities adequate?				
Спят ли дети в одной кровати с другим ребенком?<br>Do children share beds with one-another?				
Доступен ли транспорт для отправки пациентов?<br>Is transport available to send referrals?				

Есть ли следующее оборудование в вашей больнице:
Is the following equipment available in your hospital:

		Всегда Always	Зачастую Mostly	Редко Rarely	Никогда Never
Кислород?<br>Указать источник:<br>Oxygen?<br>Indicate source:	- кислородный баллон<br>(oxygen cylinder)<br>- концентратор кислорода<br>(oxygen concentrator)<br>- централизованное снабжение<br>(central supply)				
Расходомеры для кислорода?<br>Flow-meters for oxygen?					
Монитор насыщения кислородом?<br>Oxygen saturation monitor?					
Оборудование для подачи кислорода?<br>Equipment for the administration of oxygen?					

в/в системы с камерами для использования в педиатрии? (бюретки)<br>IV-giving sets with chambers for paediatric use? (burette)				
Бабочки и/или канюли детского размера?<br>Butterflies and/or cannulas of paediatric size?				
Назогастральные трубки детского размера?<br>NG-tubes, paediatric size?				
Оборудование для внутрикостного введения жидкости?<br>Equipment for intra-osseous fluid administration?				
Отсос?<br>Suction equipment?				
Небулайзеры для введения сальбутамола?<br>Nebulisers for administration of salbutamol?				
Спейсеры с масками для приема дозированного (аэрозоль) сальбутамола?<br>Spacers with masks for administration of metered doses (spray) of salbutamol?				
Функционирующее рентгеновское оборудование?<br>Functional X-ray equipment?				
Стетоскопы?<br>Stethoscopes?				
Термометры?<br>Thermometers?				

КОНТРОЛЬНЫЙ СПИСОК НАБЛЮДЕНИЙ: все проверки
OBSERVATIONAL CHECK LIST: all inspection

Затрудненное дыхание/Острое респираторное заболевание
Breathing difficulty/Acute respiratory illness

1) существуют ли письменные инструкции для оценки ребенка с затрудненным дыханием/острым респираторным заболеванием? ДА / НЕТ
Are there written guidelines available for assessing the child with breathing difficulty/Acute respiratory illness? YES / NO
If yes which are available?
Если да, то которые есть в наличии?

Настенный плакат ИВБДВ<br>IMCI Wallchart		Флипчарт ИВБДВ<br>IMCI flipchart	
Местные клинические<br>Протоколы<br>Local guidelines		Другое (опишите)<br>Other (describe):	

**Посмотрите и запишите наличие следующих вещей в палате:**<br>**Look for and record presence of the following on the ward:**	Да/Нет<br>Y/N
Мешок Амбу и маски различных размеров<br>Ambu-bag and masks of different sizes?	
Весы для новорожденных?<br>Scales for newborns?	
Весы для детей?<br>Scales for children?	
Небулайзеры?<br>Nebulizers?	
Спейсеры для управления ингаляцией отмеренной дозы лекарства?<br>Spacers for inhalation medicine metered dose administration?	
Термометры?<br>Thermometers?	
Стетоскопы?<br>Stethoscopes?	

**Противоастматические препараты (из списка основных лекарственных средств ВОЗ):**<br>**Antiasthmatic medicine (from WHO essential drugs list):**	Да/нет<br>Y/N
Будесонид для ингаляций (аэрозоль): (Budesonide, Inhalation (aerosol))	
1. 100 мкг на дозу; (100 micrograms per dose)	
2. 200 мкг на дозу; (200 micrograms per dose)	
Сальбутамол: (Salbutamol)	
1. Раствор для инъекций, 50 микрограмм (в виде сульфата) / мл в 5-мл ампуле. (Injection, 50 micrograms (as sulfate)/mL in 5-mL ampoule)	
2. Дозированный ингалятор (аэрозоль): 100 микрограмм (в виде сульфата) на дозу.	

(Metered dose inhaler (aerosol): 100 micrograms (as sulfate) per dose)<br>3. Дыхательный раствор для использования через небулайзер: 5 мг (в виде сульфата) / мл. (Respirator solution for use in nebulizers: 5 mg (as sulfate)/mL)<br>4. Пероральный Сальбутамол (не по перечню основных лекарственных средств) Oral Salbutamol (not on essential drugs list)	
Другие ингаляционные кортикостероиды:______________________________________<br>(Other inhaled cortico-steroid)	
Другие бета-2 агонисты короткого действия:____________________________________<br>(Other short acting beta-2 agonist)	

**Проверить палату и ведение всей территории медучреждения на наличие клинических руководств, книг, буклетов и плакатов<br>Inspect the ward and reference areas of the health facility for GUIDELINE books, booklets and posters**		
**Клинические руководства и плакаты<br>Guidelines and posters**	Год издания<br>Year of publication	
Список плакатов в палате<br>List of posters in the ward		
Список клинических руководств в палате<br>List of guideline books in the ward		

Список клинических руководств и плакатов в больничной библиотеке или контрольном помещении<br>List guidelines and posters in the hospital library or reference room		

Приложение G: Форма 5 Беседа со старшими сотрудниками медучреждения первичного звена
Appendix G: Form 5 Interview with senior staff from first level referral facility

Получает направления от____Сторона Fresh Air
Receives referrals from ____ Fresh Air sites

ПОЛУЧЕННЫЕ ДАННЫЕ ПО РЕЗУЛЬТАТАМ БЕСЕДЫ С ОТВЕТСТВЕННЫМИ СТАРШИМИ СОТРУДНИКАМИ
DATA OBTAINED BY INTERVIEW WITH THE RESPONSIBLE SENIOR STAFF

**Информация предоставлена (наименование должности)<br>Information Provided by (Title of post)**	**Дата<br>Date**

Введение:
Introduction:

Цель – описать ресурсы, имеющиеся в учреждении.
The purpose is to describe the resources available in the facility.

Общая информация (если неприменимо, указывают на Н/П):
General information (if not applicable, indicate by N/A):

Количество коек в педиатрическом отделении/
Number of beds in the paediatric department/
палата? Количество детских кроваток для новорожденных/младенцев
ward? Number of cots for neonatal / young infant
приемная? Ежегодное число родов в больнице?
admissions?Annual number of deliveries at the hospital?

койки		(beds)
кроватки		(cots)
ежегодно		(per year)

What is the **actual** (average) number of staff at the hospital and in the paediatric ward(s):

	Мед Учреждения (все отд-я)	Paediatric dept/ward	No. of unfilled posts?
Педиатры? (Paediatricians)	*******		
Семейные врачи? (Doctors (MOs))			
Мед. ассистенты? (Medical assistants (COs))			
Медсестры в дневную смену? (Nurses per dayshift (NDs))			
Медсестры в ночную смену? (Nurses during the nightshift? (NNs))			
Вспомогательный персонал в дневную смену? (Auxiliary staff per dayshift (ADs))			
Вспомогательный персонал в ночную смену? (Auxiliary staff during the nightshift (ANs))			

Доступные условия и удобства (есть=отмечаете, нет=оставляете пустым)
Services and facilities available: (tick=yes, blank=no)

	Всегда Always	Зачастую Mostly	Редко Rarely	Никогда Never
Есть ли электричество в вашем медучреждении? Do you have electricity at your health facility?				
Есть ли проточная вода? Do you have running water?				
У вас есть мыло и/или дезинфицирующее средство для мытья рук в детском отделении? Do you have soap and /or disinfectant for hand-washing in the paediatric ward?				
Являются ли процедуры мытья и туалетные удобства адекватными для пациента? Are the patient washing and toilet facilities adequate?				
Спят ли дети в одной кровати с другим ребенком? Do children share beds with one-another?				
Доступен ли транспорт для отправки пациентов? Is transport available to send referrals?				

Есть ли следующее оборудование в вашей больнице:
Is the following equipment available in your hospital:

		Всегда Always	Зачастую Mostly	Редко Rarely	Никогда Never
Кислород? Указать источник: Oxygen? Indicate source:	- кислородный баллон (oxygen cylinder)<br>- концентратор кислорода (oxygen concentrator)<br>- централизованное снабжения (central supply)				
Расходомеры для кислорода? Flow-meters for oxygen?					
Монитор насыщения кислородом? Oxygen saturation monitor?					
Оборудование для подачи кислорода? Equipment for the administration of oxygen?					

в/венные системы с камерами для использования в педиатрии? (бюретки)<br>IV-giving sets with chambers for paediatric use? (burette)				
Бабочки и/или канюли детского размера?<br>Butterflies and/or cannulas of paediatric size?				
Назогастральные трубки детского размера?<br>NG-tubes, paediatric size?				
Оборудование для внутрикостного введения жидкости?<br>Equipment for intra-osseous fluid administration?				
Оборудование для отсоса ?<br>Suction equipment?				
Небулайзеры для введения сальбутамола?<br>Nebulisers for administration of salbutamol?				
Спейсеры с масками для приема дозированного (аэрозоль) сальбутамола?<br>Spacers with masks for administration of metered doses (spray) of salbutamol?				
Функционирующее рентгеновское оборудование?<br>Functional X-ray equipment?				
Стетоскопы?<br>Stethoscopes?				
Термометры?<br>Thermometers?				

КОНТРОЛЬНЫЙ СПИСОК НАБЛЮДЕНИЙ: все проверки
OBSERVATIONAL CHECK LIST: all inspection

Затрудненное дыхание/Острое респираторное заболевание
Breathing difficulty/Acute respiratory illness

1) существуют ли письменные инструкции для оценки ребенка с затрудненным дыханием/острым респираторным заболеванием? ДА / НЕТ
Are there written guidelines available for assessing the child with breathing difficulty/Acute respiratory illness? YES / NO
If yes which are available?
Если да, то которые есть в наличии?

Настенный плакат ИВБДВ<br>IMCI Wallchart		Флипчарт ИВБДВ<br>IMCI flipchart	
Местные клинические Протоколы<br>Local guidelines		Другое (опишите)<br>Other (describe):	

**Посмотрите и запишите наличие следующих вещей в палате:**<br>**Look for and record presence of the following on the ward:**	Да/Нет<br>Y/N
Мешок Амбу <u>и</u> маски различных размеров<br>Ambu-bag <u>and</u> masks of different sizes?	
Весы для новорожденных?<br>Scales for newborns?	
Весы для детей?<br>Scales for children?	
Небулайзеры?<br>Nebulizers?	
Спейсеры для управления ингаляцией отмеренной дозы лекарства?<br>Spacers for inhalation medicine metered dose administration?	
Термометры?<br>Thermometers?	
Стетоскопы?<br>Stethoscopes?	

**Противоастматические препараты (из списка основных лекарственных средств ВОЗ):**<br>**Antiasthmatic medicine (from WHO essential drugs list):**	Да/нет<br>Y/N
Будесонид для ингаляций (аэрозоль): (Budesonide, Inhalation (aerosol))	
1. 100 мкг на дозу; (100 micrograms per dose)	
2. 200 мкг на дозу; (200 micrograms per dose)	
Сальбутамол: (Salbutamol)<br>1. Раствор для инъекций, 50 микрограмм (в виде сульфата) /	

мл в 5-мл ампуле.<br>(Injection, 50 micrograms (as sulfate)/mL in 5-mL ampoule)<br>2. Дозирующий ингалятор (аэрозоль): 100 микрограмм (в виде сульфата) на дозу.<br>(Metered dose inhaler (aerosol): 100 micrograms (as sulfate) per dose)<br>3. Дыхательный раствор для использования через небулайзер: 5 мг (в виде сульфата) / мл. (Respirator solution for use in nebulizers: 5 mg (as sulfate)/mL)<br>4. Пероральный Сальбутамол (не по перечню основных лекарственных средств)<br>Oral Salbutamol (not on essential drugs list)	
Другие ингаляционные кортикостероиды:____________________________________<br>(Other inhaled cortico-steroid)	
Другие бета-2 агонисты короткого действия:___________________________________<br>(Other short acting beta-2 agonist)	

(UOI) |__|__|-|__|__|-|__|__| **(UPI) |__|__|** **(UCI) |__|__|__|**

MẪU SÀNG LỌC

<table>
<tr><td>Mã số bệnh nhân</td><td colspan="2">__________</td></tr>
<tr><td>Mã người thu thập số liệu</td><td colspan="2">|__|__|</td></tr>
<tr><td>Ngày nghiên cứu</td><td colspan="2">_____/_____/_____ (DD/MM/YY)</td></tr>
<tr><td>Mã cơ sở y tế</td><td colspan="2">|__|__|</td></tr>
<tr><td>Quốc gia</td><td>1. Greece
2. Kyrgystan</td><td>3. Vietnam
4. Uganda</td></tr>
<tr><td>Tháng tuổi của trẻ: _____ tháng</td><td colspan="2">Ngày tháng năm sinh của trẻ: _____/_____/_____</td></tr>
<tr><td>Giới tính của trẻ</td><td colspan="2">1. Nam 2. Nữ</td></tr>
<tr><td colspan="3"><u>Triệu chứng chính</u> đến khám

1. Ho / khó / thở nhanh / khò khè
2. Thay đổi trong ý thức / thờ ơ
3. Co giật
4. Nôn tất cả mọi thứ
5. Không chịu ăn hoặc uống
6. Sốt / sốt rét
7. Tiêu chảy / nôn
8. Vấn đề về tai
9. Sởi
10. Vấn đề về dinh dưỡng hay ăn uống
11. Khác______________________________</td></tr>
<tr><td>Trước khi đưa trẻ đến khám vì bệnh lần này, Anh/chị có điều trị gì cho trẻ chưa?</td><td colspan="2">1. Có 2. Không</td></tr>
<tr><td colspan="3">Nếu có, cách điều trị là gì?

1. Mua thuốc ngoài tiệm thuốc Tây
2. Đến phòng khám tư
3. Trạm y tế
4. Tự điều trị tại nhà theo kinh nghiệm
5. Khác______________________________</td></tr>
</table>

BẢNG KIỂM QUAN SÁT NHÂN VIÊN Y TẾ

Mục	Đáp án chọn lựa	Hướng dẫn
**Mã người thu thập số liệu**	|___|___|	2 chữ số gắn cho mỗi người thu thập số liệu.
**Mã cơ sở y tế**	|___|___|	2 chữ số gắn cho mỗi cơ sở y tế
**Số thứ tự quan sát**	|___|___|	2 chữ số: Số cuộc quan sát gắn với mỗi quan sát theo thứ tự quan sát
**Mã quan sát duy nhất (UOI)**	|___|___|-|___|___|-|___|___|	2-mã số người thu thập +<br>2-Mã số CSYT +<br>2-Mã số thứ tự cuộc quan sát<br>Chép mã quan sát duy nhất này vào mỗi trang của bảng quan sát
**Mã nhân viên y tế (UPI)**	|___|___|	Gắn 2 mã số cho mỗi NVYT được quan sát bắt đầu từ 01-10
**Mã bệnh nhân duy nhất (UCI)**	|___|___|___|	Gắn 2 mã số cho mỗi BN được quan sát lần lượt từ 01-210
**Ngày khám(DOV)**	|___|___| / |___|__ / |___|___|	**Nhập ngày tháng theo format:**<br>(DD/ MM / YY)

(UOI) |__|__|-|__|__|-|__|__| (UPI) |__|__| (UCI) |__|__|__|

PHẦN 1: THÔNG TIN DÂN SỐ

Câu	Nội dung	Trả lời	Mã hóa
1	Tên nhân viên y tế	………………………………………	
2	Nhân viên y tế được quan sát là?	1. Bác sĩ nhi khoa<br>2. Bác sĩ đa khoa<br>3. Bác sĩ gia đình<br>4. Y sĩ<br>5. Bác sĩ y học cổ truyền<br>6. Khác:_________________________	
3	Học vấn cao nhất?	1. Thạc sĩ<br>2. Tiến sĩ<br>3. Chuyên khoa I<br>4. Chuyên khoa II<br>5. Khác:_________________________	
4	Thời gian công tác	________________ tháng	
5	Thời gian bắt đầu thăm khám	Giờ__________Phút_________________	
6	Thời gian kết thúc thăm khám	Giờ__________Phút_________________	

(UOI) |__|__|-|__|__|-|__|__| (UPI) |__|__| (UCI) |__|__|__|

MẪU 1: QUAN SÁT HỎI BỆNH CỦA NHÂN VIÊN Y TẾ

Câu	Nội dung	Trả lời
**Bác sĩ có hỏi những vấn đề sau hay không?**		
**1**	***Những than phiền a?***	1. Có 2. Không
1	Người chăm sóc trả lời về những triệu chứng hiện mắc a<br><br>(Nhiều chọn lựa)	1. Ho<br>2. Khó thở<br>3. Khò khè<br>4. Thở có tiếng<br>5. Thở nhanh<br>6. Tức ngực<br>7. Sốt<br>8. Khác: ______________________
**2**	***Những than phiền khác?***	1. Có 2. Không
2	Trả lời về những than phiền khác<br>(từ người chăm sóc trẻ)	--
**3**	***Số ngày trẻ bị bệnh***	1. Có 2. Không
3	BN trả lời số ngày trẻ bị bệnh	________ ngày
**4. NVYT có hỏi về những thông tin sau hay không?**		
**4a**	***Số ngày trẻ ho?***	1. Có 2. Không
4a	Thời gian trẻ ho (tự nói/trả lời)	________ ngày
**4b**	***Ho vào ban đêm hay sáng sớm***	1. Có 2. Không
4b	Ho vào ban đêm hay sáng sớm (tự nói/trả lời)	--
**4c**	***Trẻ có ho lặp đi lặp lại?***	1. Có 2. Không
4c	Trẻ có ho lặp đi lặp lại? (tự nói/trả lời)	--
**4d**	***Khó thở trong lần bệnh này***	1. Có 2. Không
4d	Khó thở trong lần bệnh này (tự nói/trả lời)	--
**4e**	***Khó thở lặp đi lặp***	1. Có 2. Không
4e	Khó thở lặp đi lặp lại (tự nói/trả lời)	--
**4f**	***Thở khò khè trong lần bệnh này***	1. Có 2. Không
4f	Thở khò khè trong lần bệnh này	--
	Hỏi về tác nhân gây kích thích?	1. Có 2. Không

**5. Các triệu chứng trên (ho, khò khè, khó thở) xuất hiện hoặc trầm trọng hơn khi có sự hiện diện của các yếu tố sau (lưu ý: câu hỏi này có thể theo sau các câu hỏi trên)**		
**5a**	***Môi trường bụi***	1. Có 2. Không
5a	Môi trường bụi (tự nói/trả lời)	1. Có 2. Không
**5b**	**Nhiễm virut cúm đường hô hấp/cảm cúm**	1. Có 2. Không
5b	Nhiễm virut cúm đường hô hấp (tự nói/trả lời)	1. Có 2. Không
**5c**	***Khói sinh khối***	1. Có 2. Không
5c	Khói sinh khối (tự nói/trả lời)	1. Có 2. Không
**5d**	***Khói thuốc lá***	1. Có 2. Không
5d	Khói thuốc lá (tự nói/trả lời)	1. Có 2. Không
**5e**	***Hóa chất bình phun/xịt***	1. Có 2. Không
5e	Hóa chất bình phun/xịt (tự nói/trả lời)	1. Có 2. Không
**5f**	***Thay đổi về thời tiết (lạnh)***	1. Có 2. Không
5f	Thay đổi về thời tiết (lạnh) (tự nói/trả lời)	1. Có 2. Không
**5g**	***Vật nuôi trong nhà (chó, mèo)***	1. Có 2. Không
5g	Vật nuôi trong nhà (chó, mèo) (tự nói/trả lời)	1. Có 2. Không
**5h**	***Trong hoặc sau khi tập thể dục***	1. Có 2. Không
5h	Trong hoặc sau khi tập thể dục (tự nói/trả lời)	1. Có 2. Không
**5i**	***Tác nhân khác được chú ý do người chăm sóc NCS***	1. Có 2. Không
5i	Các tác nhân khác được chú ý do NCS (tự nói/trả lời)	1. Có 2. Không
	Nếu có, cụ thể:	---------------------------------------
	**Tiền sử dị ứng của cha/mẹ trẻ?**	1. Có 2. Không

6. Nhân viên y tế có hỏi về các yếu tố sau?<br>(Lưu ý hỏi BN thông tin cụ thể khi câu trả lời 'Có')		
**6a**	***Tiền sử dị ứng của trẻ***	1. Có 2. Không
6a	Tiền sử dị ứng của trẻ (BN tự nói/trả lời)	1. Có<br>2. Không 3. Khác: ____________
**6b**	***Tiền sử gia đình bị bệnh hen suyễn***	1. Có 2. Không
6b	Tiền sử gia đình bị bệnh hen suyễn<br>(BN tự nói/trả lời)	1. Có<br>2. Không 3. Khác: ____________
**6c**	***Tiền sử gia đình bị di ứng***	1. Có 2. Không
6c	Tiền sử gia đình bị di ứng (BN tự nói/trả lời)	1. Có<br>2. Không 3. Khác: ____________
Hỏi về uống thuốc trước đây/ trước tới khám		1. Có 2. Không
BS xem lại những lưu ý trên các toa thuốc trước đây?		1. Có 2. Không
7. Từng dùng các loại thuốc điều trị trước đây		
**6d.i**	**Dùng Salbutamol (uống, phun khí dung, hít)**	1. Có 2. Không
6d.i	Dùng salbutamol (uống, phun khí dung, hít)<br>(BN tự nói/trả lời)	1. Uống 4. Không<br>2. Phun khí dung 5. Khác:__________<br>3. Hít
**6d.ii**	***Hỏi về dùng Corticosteroids***<br>***(uống, chích, phun khí dung, hít)***	1. Có 2. Không
6d.ii	Dùng thuốc Corticosteroids<br>(BN tự nói/trả lời)	1. Uống 4. Không<br>2. Chích 5. Khác:__________<br>3. Hít
**6diii**	***Dùng kháng sinh***	1. Có 2. Không
6diii	Loại thuốc kháng sinh sử dụng<br>(BN tự nói/trả lời)	________________________________
**6d.iv**	**Sử dụng bất kì một loại thuốc ho nào**	1. Có 2. Không
6d.iv	Thuốc ho sử dụng (BN tự nói/trả lời)	---
**6d.v**	**Sử dụng thuốc ho thảo dược**	1. Có 2. Không
6d.v	Sử dụng thuốc ho thảo dược (tự nói/trả lời)	---

**6d.vi**	***Dùng thuốc Montelukast***	1. Có 2. Không
6d.vi	Dùng thuốc Montelukast (tự nói/trả lời)	---
**6d.vii**	***Dùng thuốc Antihistamin***	1. Có 2. Không
6d.vii	Dùng thuốc Antihistamin (tự nói/trả lời)	---
Hỏi về những lần khám bệnh trước với bác sĩ trước đây		

6e Những lần thăm khám trước (phòng khám tư, nhà thuốc cho cùng loại bệnh này)				
6e.i	Hỏi về số lần khám trước đây (BS hỏi)	Có<br>Không	1<br>2	
6e.i	Số lần khám trước đây (BN tự nói/trả lời)	____________lần		
6e.ii	Hỏi về sử dụng thuốc (BS hỏi)	Có<br>Không	1<br>2	
6e.ii	Thuốc đã được sử dụng (BN tự nói/trả lời)	___________________________________		
7 Sốt				
7a	Hỏi trẻ có sốt không? (BS hỏi)	Có<br>Không	1<br>2	
7a	Sốt (BN tự nói/trả lời)	Có<br>Không	1<br>2	
7b	Hỏi về số ngày sốt? (BS hỏi)	Có<br>Không	1<br>2	
7b	Số ngày sốt (BN tự nói/trả lời)	___________________ngày		

(UOI) |__|__|-|__|__|-|__|__| (UPI) |__|__| (UCI) |__|__|__|

PHẦN 3: TRIỆU CHỨNG LÂM SÀNG

Câu	Nội dung	Trả lời	Mã hóa	Ghi chú
8	Cân nặng	Có<br>Không	1<br>2	
8	Cân nặng của trẻ	________________kg		
9	Hỏi trẻ có đau hoặc không tỉnh táo hay không?	Có<br>Không	1<br>2	
9	Trẻ đau hoặc không tỉnh táo	Trẻ có đau thực thể<br>Trẻ không tỉnh táo	1<br>2	
10	Trẻ thở khò khè nghe rõ	Có<br>Không	1<br>2	
11	Nhiệt độ	Có<br>Không	1<br>2	
11	Nhiệt độ (đo/cảm nhận, sờ)	____________________ độ		
12	Độ bão hòa oxy (SPO_2)	Có<br>Không	1<br>2	
12	Độ bão hòa oxy (SPO_2)	______________________		
	**HỆ HÔ HẤP**			
14	Trẻ có được khám hệ hô hấp không?	Có<br>Không	1<br>2	
15	Trẻ được đếm nhịp thở không?	Có<br>Không	1<br>2	
15	Nhịp thở (hỏi bác sĩ lúc BS khám xong)	____________________ /phút		
16	Trẻ có lồng ngực lõm không?	Có<br>Không	1<br>2	
17	NVYT có sử dụng ống nghe không?	Có<br>Không	1<br>2	

17a	Kết quả từ ống nghe	Bình thường<br>Nổ lốp đốp<br>Tiếng nổ<br>Thở khò khè<br>Nghe thấy tiếng rales<br>Tiếng ngáy<br>Hô hấp phế quản (bronchial respiration)<br>Ngực trong (silent chest)<br>Khác (ghi rõ)<br>____________________	1<br>2<br>3<br>4<br>5<br>6<br>7<br>8<br>9	

PHẦN 4: GIAO TIẾP VỚI NGƯỜI CHĂM SÓC

Câu	Nội dung	Trả lời	Mã hóa	Ghi chú
NVYT tế có truyền thông cho những người chăm sóc về các điều dưới đây không?				
18a	Kết quả lâm sàng	Có<br>Không	1<br>2	
18a	Kết quả lâm sàng	____________________		
18b	Chẩn đoán sơ bộ	Có<br>Không	1<br>2	
18b	Chẩn đoán sơ bộ	Viêm đường hô hấp trên<br>Nhiễm virut<br>Viêm phế quản<br>Viêm phổi<br>Hen/Suyễn<br>Viêm họng<br>Ho gà<br>Lao<br>Khác (ghi rõ)<br>____________________	1<br>2<br>3<br>4<br>5<br>6<br>7<br>8<br>9	

18c	Nguyên nhân có thể dẫn tới bệnh	Có<br>Không	1<br>2	
18c	Nguyên nhân có thể dẫn tới bệnh	________________		
18d	Điều trị	Có<br>Không	1<br>2	
18d	Điều trị	________________		
18e	Liều lượng thuốc	Có<br>Không	1<br>2	
18e	Liều lượng thuốc	________________		
18f	Thời gian sử dụng thuốc	Có<br>Không	1<br>2	
18f	Thời gian sử dụng thuốc	________________		
18g	Công dụng/cơ chế của thuốc?	Có<br>Không	1<br>2	
18g	Công dụng/cơ chế của thuốc	________________		
18h	Các tác dụng phụ của thuốc	Có<br>Không	1<br>2	
18h	Các tác dụng phụ của thuốc	________________		
18i	Tầm quan trọng của việc tuân thủ điều trị	Có<br>Không	1<br>2	
18i	Tầm quan trọng của việc tuân thủ điều trị	________________		
18j	Khi nào quay trở lại tái khám	Có<br>Không	1<br>2	
18j	Khi nào quay trở lại tái khám	________________		

Đối với trẻ bị suyễn nặng, các nhân viên y tế có trao đổi về những vấn đề sau đây				
19a	Nguyên nhân	Có<br>Không	1<br>2	
19b	Tầm quan trọng của việc trành tiếp xúc với những tác nhân	Có<br>Không	1<br>2	
19c	Phải làm gì trong trường hợp trẻ lên cớm khó thở khò khè	Có<br>Không	1<br>2	
19d	Sử dụng các loại thuốc cấp cứu?	Có<br>Không	1<br>2	
19e	Các loại thuốc cấp cứu sử dụng như thế nào? (hít nếu có)	Có<br>Không	1<br>2	
19f	Khi nào cần đưa đến cơ sở y tế	Có<br>Không	1<br>2	
19g	NVYT có tư vấn những chẩn đoán khác để đánh giá thêm hay không?	Có<br>Không	1<br>2	

Chú thích thêm
………………………………………………………………………………………………
………………………………………………………………………………………………
………………………………………………………………………………………………
………………………………………………………………………………………………
…………

(UOI) |__|__|-|__|__|-|__|__| (UPI) |__|__| (UCI) |__|__|__|

MẪU 2 PHỎNG VẤN NGƯỜI CHĂM SÓC

Mục	Đáp án chọn lựa	Hướng dẫn
**Mã người thu thập số liệu**	|__|__|	2 chữ số gắn cho mỗi người thu thập số liệu.
**Mã trạm y tế**	|__|__|	Enter pre-assigned number that corresponds to the facility
**Số thứ tự quan sát**	|__|__|	2 chữ số: Số cuộc quan sát gắn với mỗi quan sát theo thứ tự quan sát
**Mã quan sát duy nhất (UOI)**	|__|__|-|__|__|-|__|__|	2-mã số người thu thập +<br>2-Mã số TYT +<br>2-Mã số thứ tự cuộc quan sát<br>Chép mã quan sát duy nhất này vào mỗi trang của bảng quan sát
**Mã nhân viên y tế (UPI)**	|__|__|	Gắn 2 mã số cho mỗi NVYT được quan sát bắt đầu từ 01-10
**Mã bệnh nhân duy nhất (UCI)**	|__|__|__|	Gắn 2 mã số cho mỗi BN được quan sát lần lượt từ 01-100
**Ngày khám(DOV)**	|__|__| / |__|__ / |__|__|	**Nhập ngày tháng theo format:**<br>(DD/ MM / YY)
**SĐT người chăm sóc**		
**Đặc điểm dân số - xã hội:**		
**Học vấn cao nhất của chị? (Mẹ của trẻ)**		Lớp ________________
**Năm sinh của người mẹ**		__________________
**Trẻ có sinh đôi hay không?**		1. Có 2. Không
**Trẻ có bao nhiêu anh chị em ruột?**<br>**(Không tính trẻ)**		_______________người

(UOI) |__|__|-|__|__|-|__|__| (UPI) |__|__| (UCI) |__|__|__|

PHẦN 1:

STT	Câu hỏi	Câu trả lời	Ghi chú
**1. NVYT có trao đổi với anh/chị những vấn đề sau:**			
1a	Kết quả chẩn đoán?	1. Có	2. Không
1b	Nguyên nhân có thể dẫn tới bệnh?	1. Có	2. Không
1c	Loại thuốc mà bác sỹ kê đơn?	1. Có	2. Không
1d	Uống thuốc như thế nào?	1. Có	2. Không
1e	Liều lượng uống thuốc?	1. Có	2. Không
1f	Số lần uống thuốc?	1. Có	2. Không
1g	Thời gian điều trị?	1. Có	2. Không
1h	Khi nào cần nhập viện ngay lập tức?	1. Có	2. Không
1i	Thời gian tái khám?	1. Có	2. Không
**2. Đối với trẻ nghi ngờ bị hen xuyễn NVYT có trao đổi với anh/chị những vấn đề sau:**			
2a	Nguyên nhân?	1. Có	2. Không
2b	Phải làm gì khi xuất hiện cơn khó thở, khò khè?	1. Có	2. Không
2c	Các loại thuốc anh/chị nên cho bé uống nếu bé bắt đầu cơn khó thở, khò khè?	1. Có	2. Không
2d	Làm thế nào để kiểm soát cơn cơn khó thở, khò khè đó?	1. Có	2. Không

(UOI) |__|__|-|__|__|-|__|__| **(UPI) |__|__|** **(UCI) |__|__|__|**

PHẦN 2:

STT	Câu hỏi	Câu trả lời	Ghi chú
3a	Trong nhà anh/chị có ai hút thuốc không?	1. Có	2. Không
4a	Con của bạn có tiếp xúc với khói thuốc lá hay khói bếp khi ở nhà hay không? ***(Nếu không, chuyển qua 4d)***	1. Có	2. Không
4b	Thời gian trẻ tiếp xúc với khói đó trong bao lâu?	____________________tháng	
4c	Thời gian trung bình mỗi ngày trẻ tiếp xúc với khói đó?	____________________giờ	
4d	Chị (mẹ của đứa trẻ) có tiếp xúc với khói thuốc lá hay khói bếp trong thai kì không?	1. Có	2. Không
4f	Con của anh/chị có từng sử dụng thuốc dạng hơi/xịt trước đó hay không?	1. Có	2. Không
**5a**	**Trẻ có tiền sử ho/khó thở hay không? (*Nếu không chuyển qua câu 7*)**	1. Có	2. Không
5b	Số lần trẻ có tiền sử ho/khó thở?	_____________________lần	
5c	Đợt ho kéo dài hơn 14 ngày?	1. Có	2. Không

PHẦN 3:
GHI CHÉP THÔNG TIN TỪ HỒ SƠ BỆNH ÁN CỦA BỆNH NHÂN

Kiểm tra hồ sơ bệnh án của bệnh nhân và ghi lại như sau:

1a	Có ghi nhiệt độ của trẻ hay không	1. Có	2. Không
1b	Nhiệt độ	___________________________ ^{0}C	
2a	Có ghi nhịp thở của trẻ hay không	1. Có	2. Không
2b	Nhịp thở	________________________ nhịp/phút	
3a	Khám phổi có ghi nhận gì không?	1. Có	2. Không

	(Nếu không chuyển qua câu 4)	
3b	Nếu có, ghi nhận là gì? ***(câu hỏi nhiều lựa chọn)***	1. Bình thường<br>2. Nổ lốp đốp<br>3. Tiếng nổ<br>4. Thở khò khè<br>5. Nghe thấy tiếng rales<br>6. Tiếng ngáy<br>7. Hô hấp phế quản<br>8. Ngực trong (silent chest)<br>9. Khác (ghi rõ)<br>________________________________<br>10. Không ghi nhận được thông tin
3c	Nghe phổi khác	________________________________
4	Chẩn đoán ***(chọn nhiều đáp án)***	1. Nhiễm virus<br>2. Viêm phế quản<br>3. Viêm phổi<br>4. Viêm tiểu phế quản<br>5. Bệnh hen suyễn<br>6. Viêm hầu họng<br>7. Ho gà<br>8. Lao<br>9. Không có chẩn đoán<br>10. Khác (ghi rõ)<br>________________________________<br>11. Không ghi nhận được thông tin
5	Chỉ định điều trị?	1. Kháng sinh

	(Chọn nhiều đáp án)	2. Thuốc tiêu đờm<br>3. Thuốc giãn phế quản<br>4. Sterioid dạng uống<br>5. Sterioid dạng hít<br>6. Sterioid dạng tiêm<br>7. Thuốc kháng virut<br>8. Lao<br>9. Không sử dụng thuốc<br>10. Khác (ghi rõ)<br><br>________________________________
6	Có nghi ngờ bệnh suyễn không?	1. Có 2. Không
7	Nếu ho hoặc khó thở, sử dụng các thuốc giãn phế quản theo quy định và xác định	1. Có 2. Không
8	Nếu sử dụng thuốc giãn phế quản, tác dụng của thuốc có hay không?	1. Có 2. Không
9	Hít corticosteroid-ICS (tên)	________________________________
10	Uống corticosteroid-ICS (tên)	________________________________
11	Tiêm corticosteroid (tên)	________________________________
12	Thuốc kháng sinh (tên)	________________________________
13	Phun khí dung	1. Có 2. Không
14a	Đứa trẻ có bất kì điều trị hô hấp (BD, ICS) được chỉ định, mà cần cung cấp thiết bị nào dạng hít/xịt mới hay không? **(*Nếu không chuyển qua câu 15*)**	1. Có 2. Không
14b	Đứa trẻ có bất kì điều trị hô hấp (BD, ICS) được chỉ định, mà cần cung cấp thiết bị	

	nào dạng hít/xịt mới hay không?	***(Ghi rõ)***__________________________
15	Chỉ định điều trị khác (tên)	______________________________
16	Có chỉ định cận lâm sang hay không?<br>***(Nếu không chuyển qua câu 17)***	1. Có 2. Không
16a	Chỉ định cận lâm sàng?	1. Chụp X-ray<br>2. Phân tích máu<br>3. Xét nghiệm nước tiểu<br>4. Khác (ghi rõ)<br>_____________________________
17	Gửi đề kiểm tra	_____________________________
18	Chuyển tuyển hoặc điều trị tại nhà	1. Bác sĩ chuyên khoa hô hấp<br>2. Bác sĩ nội trú<br>3. Cấp cứu<br>4. Phun khí dung
18a	Chuyển tuyển hoặc điều trị tại nhà	_____________________________
19	Sắp xếp lịch theo dõi?	1. Có 2. Không
19a	Sắp xếp lịch theo dõi?	_____________________________

(UOI) |__|__|-|__|__|-|__|__| **(UPI)** |__|__| **(UCI)** |__|__|__|

MẪU 3: MẪU THEO DÕI SAU 5 NGÀY

Tên người chăm sóc: ………………………Tên trẻ: ……………………

Người giám sát: …………………………………………………….

Ngày gọi lại: ______/_____/2016

Địa điểm: ……………………………………………………….

Theo dõi 5 ngày:

Sốt (Y/N): ______________________________

Vẫn còn khó thở (Y/N): _______________________

Tham gia theo dõi định kì tại HC (Y/N): ____________

Nhập viện (Y/N): _______________

Trẻ sống (Y/N): _____________

1	Trẻ còn sốt hay không?	2. Có<br>3. Không
2	Trẻ còn khó thở hay không?	1. Có<br>2. Không
3	Trẻ có tiếp tục tái khám tại cở sở hay không?	1. Có<br>2. Không
4	Trẻ có nhập viện hay không?	1. Có<br>2. Không
5	Trẻ còn sống hay không?	2. Có<br>3. Không

(UOI) |__|__|-|__|__|-|__|__| **(UPI) |__|__|** **(UCI) |__|__|__|**

DATA OBTAINED BY INTERVIEW WITH RESPONSIBLE SENIOR STAFF

Thông tin này được cung cấp bởi<br>Information Provided by (Title of post)	Ngày<br>Date

IMCI (**hoạt động Lồng ghép chăm sóc trẻ bệnh) trong cơ sở y tế**
IMCI within the health facility:
Có một người đầu mối chung/ rành về IMCI (hoạt động Lồng ghép chăm sóc trẻ bệnh) trong cơ sở y tế?
Có/ Không/Không biết
Do you have a IMCI focal person within the health facility Y/N/don't know
Có bao nhiêu nhân viên y tế được tập huấn về chiến lược IMCI (hoạt động Lồng ghép chăm sóc trẻ bệnh)?
How many staff members have been trained in the IMCI strategy:_________________,
Và bao nhiêu trong số này tập huấn trong 2015
and how many of these within 2015:_________________

Có **loại hình đào tạo nhi khoa** nào **khác** được **thực hiện** tại bệnh viện **trong 2015**? Liệt kê dưới đây
Any other kind of training for paediatric staff has taken place in 2015:

1. ____
2. ____
3. ____
4. ____

Cơ sở y tế có nhận nguồn kinh phí/ vật liệu hỗ trợ nào từ nguồn khác ngoài nguồn nhà nước không?
Has the health facility received economic/ material support from other sources than the public?

1. From church organisation Y/N List:________________________________
Tổ chức nhà thờ Có/Không

2. Private NGO's Y/N List:________________________________
Tổ chức phi chính phủ Có/Không

3 Other organisations Y/N List:________________________________
Tổ chức khác Có/Không

(UOI) |__|__|-|__|__|-|__|__| **(UPI) |__|__|** **(UCI) |__|__|__|**

Giới thiệu:
Mục đích của mẫu này nhằm mô tả nguồn lực sẵn có tại cơ sở

The purpose is to describe the resources available to the facility.**Thông tin chung (nếu không áp dụng, thể hiện bằng N/A):**<br>**General information (if not applicable, indicate by N/A):**			
**Số giường** tại **khoa/phòng nhi**?<br>Number of beds in the paediatric department/ ward?			Giường<br>Beds
**Số lượng** cũi/**nôi cho trẻ sơ sinh nhập viện**?<br>Number of cots for neonatal / young infant admissions?			Cũi/nôi<br>Cots
**Số lượng sinh** tại bệnh viện **hàng năm**?<br>Annual number of deliveries at the hospital?			Hàng năm<br>Per year

Số lượng nhân viên trung bình hiện có thực tế tại bệnh viện và tại khoa nhi là bao nhiêu?<br>What is the **actual** (average) number of staff at the hospital and in the paediatric ward(s):	Số lượng tại bệnh viện/ các khoa phòng (Health facility (all dept.)		Số lượng tại khoa nhi<br>Paediatric dept/ward	No. of unfilled posts?
**Bác sĩ nhi khoa?**<br>Paediatricians?				
**Bác sĩ?**<br>Doctors? (MOs)				
**Y sĩ?**<br>Medical assistants? (COs)				
**Y tá trực ca ngày?**<br>Nurses per day shift? (NDs)				
**Y tá trực ca đêm?**<br>Nurses during the night shift? (NNs)				
**Nhân viên hỗ trợ ca ngày?**<br>Auxiliary staff per dayshift? (ADs)				

**Nhân viên hỗ trợ ca đêm?**<br>Auxiliary staff during the nightshift? (ANs)				

**Phân loại và điều trị cho trẻ em chờ được khám**<br>**Triage and Treatment (ETAT) for children waiting to be seen.**	
Có bất kỳ **hình thức phân loại bệnh nhân** nào khi **đến khám tại cơ sở y tế** không?<br>Is there any form of triage of patients attending the health facility	**Có/Không**<br>Y/N
**Nếu CÓ:**<br>If YES:	
**Ai chịu trách nhiệm phân loại?**<br>Who is responsible for triage?	
**Có bất kỳ hướng dẫn phân loại trong cơ sở y tế không? Nếu có hãy mô tả?**<br>Are there any guidelines on triage in the health facility? If so please describe	
**Mô tả các loại phân loại và sẽ làm gì với mỗi phân loại?**<br>Describe the triage categories and what happens to each category?	

**Các dịch vụ và phương tiện sẵn có: dấu x: có, để trống: không**<br>**Services and facilities available: (tick=yes, blank=no)**				
	Luôn luôn<br>Always	Hầu hết<br>Mostly	Thỉnh thoảng<br>Rarely	Không bao giờ<br>Never
**Có điện tại cơ sở y tế/bệnh viện?**<br>Do you have electricity at your health facility?	☐	☐	☐	☐
**Có nước máy?**<br>Do you have running water?	☐	☐	☐	☐
**Có xà phòng và/hoặc nước rửa tay diệt khuẩn trong khoa nhi?**<br>Do you have soap and /or disinfectant for hand-washing in the paediatric ward?	☐	☐	☐	☐

**Phòng giặt đồ và nhà vệ sinh có đủ không?**<br>Are the patient washing and toilet facilities adequate?	☐	☐	☐	☐
**Trẻ bệnh có nằm chung giường với trẻ khác không?**<br>Do children share beds with one-another?	☐	☐	☐	☐
**Có phương tiện vận chuyển giao thông sẵn có để chuyển bệnh nhân không?**<br>Is transport available to send referrals?	☐	☐	☐	☐

**Những thiết bị dưới đây có sẵn tại bệnh viện không?**<br>**Is the following equipment available in your hospital:**					
		Always	Often	Rarely	Never
Oxy?<br>Nguồn cung cấp:	__ oxygen cylinder<br>__ oxygen concentrator<br>__ central supply	☐	☐	☐	☐
Flow mét cho oxy?<br>Flow-meters for oxygen?		☐	☐	☐	☐
**Màn hình theo dõi bão hòa oxy?**<br>Oxygen saturation monitor?		☐	☐	☐	☐
**Thiết bị quản lý oxy?**<br>Equipment for the administration of oxygen?		☐	☐	☐	☐
**Bộ kim dẫn truyền dịch dành cho nhi?**<br>IV-giving sets with chambers for paediatric use? (burette)		☐	☐	☐	☐
**Kim bướm và hoặc ống thông kích cỡ nhi?**<br>Butterflies and/or cannulas of paediatric size?		☐	☐	☐	☐
**Ống NG (ống thông đường mũi bao tử) kích thước nhi?**<br>NG-tubes, paediatric size?		☐	☐	☐	☐
**Thiết bị quản lý dịch intra-osseous (trong xương)**<br>Equipment for intra-osseous fluid administration?		☐	☐	☐	☐
**Dụng cụ dẫn lưu?**<br>Suction equipment?		☐	☐	☐	☐
**Thiết bị phun sương cho sử dụng salbutamol?**<br>Nebulisers for administration of salbutamol?		☐	☐	☐	☐
**Buồng đệm với mặt nạ cho quản lý liều dùng Salbutamol (dạng xịt) ?**<br>Spacers with masks for administration of metered doses (spray) of salbutamol?		☐	☐	☐	☐

**Thiết bị chụp X quang chức năng?**<br>Functional X-ray equipment?	☐	☐	☐	☐

(UOI) |__|__|-|__|__|-|__|__| (UPI) |__|__| (UCI) |__|__|__|

BẢNG KIỂM QUAN SÁT
OBSERVATIONAL CHECK LIST: all inspection

Khó thở/bệnh hô hấp cấp tính

Breathing difficulty/Acute respiratory illness

1) Có **hướng dẫn có sẵn** cho việc **đánh giá trẻ khó thở/bệnh hô hấp** cấp tính không? **Có/Không**
Are there written guidelines available for assessing the child with breathing difficulty/Acute respiratory illness? YES / NO
Nếu có, cái nào dưới đây có sẵn?
If yes which are available?

IMCI (Hoạt động Lồng ghép chăm sóc trẻ bệnh) biểu đồ dán ☐   IMCI flipchart/IMCI lật ☐

Hướng dẫn nội bô/local guidline ☐   Khác (mô tả) / Other (describe):

**Tìm ghi nhận cơ sở hiện có của đơn vị:**<br><br>Look for and record presence of the following on the ward:	**Có/Không**<br><br>Yes/No
**Túi chân không hoặc mặt nạ nhiều kích thước**<br><br>Ambu-bag <u>and</u> masks of different sizes?	
**Cân dùng cho trẻ sơ sinh?**<br><br>Scales for newborns?	
**Cân dùng cho trẻ?**<br><br>Scales for children?	
**Máy phun khí dung?**<br><br>Nebulizers?	
**Buồng đệm cho quản lý sử dụng liều thuốc?**<br><br>Spacers for inhalation medicine metered dose administration?	
**Nhiệt kế?**<br><br>Thermometers?	

**Ống nghe?**<br><br>Stethoscopes?	

**Thuốc chống hen (từ danh sách thuốc thiết yếu của WHO)**<br>**Antiasthmatic medicine (from WHO essential drugs list):**	Có/Không<br>Yes/No
**Budesonide, hít (aerosol):**	
1. 100 micrograms mỗi liều;	
2. 200 micrograms mỗi liều	
**Salbutamol:**<br>1. Chích, 50 micrograms (as sulfate)/mL in 5-mL ống.	
2. Bình hít định lượng (aerosol): 100 micrograms (as sulfate) mỗi liều.	
3. Máy phun khí dung Respirator solution for use in nebulizers: 5 mg (as sulfate)/mL.	
4. Salbutamol đường uống(không có trong danh sách thuốc thiết yếu)	
**Thuốc hít cortico-steroid** khác: ______________________________	
**Chất đồng vận beta 2 tác dụng ngắn**: ___________________________	
**cortico-steroid đường uống**:______________________________	

**Inspect the ward and reference areas of the health facility for GUIDELINE books, booklets and posters**		
**Hướng dẫn và áp phích dán tường**<br><br>**Guidelines and posters**	Năm công bố	
Liệt kê các áp phích dán tường trong khoa nhi<br><br>List posters on the ward		
**Liệt kê các sách hướng dẫn trong khoa nhi**<br><br>List guideline books on the ward		

(UOI) |__|__|-|__|__|-|__|__| (UPI) |__|__| (UCI) |__|__|__|

Fresh Air Form 5: Phỏng vấn nhân viên cấp cao từ cơ sở **chuyển tuyến ban đầu**

Receives referrals from ____ Fresh Air sites
DATA OBTAINED BY INTERVIEW WITH THE RESPONSIBLE SENIOR STAFF

**Thông tin được cung cấp bởi**	**Ngày**

Giới thiệu:
Mục đích của mẫu này nhằm mô tả nguồn lực sẵn có tại cơ sở

The purpose is to describe the resources available to the facility.**Thông tin chung (nếu không áp dụng, thể hiện bằng N/A):**<br>**General information (if not applicable, indicate by N/A):**			
**Số giường tại khoa/phòng nhi?**<br>Number of beds in the paediatric department/ ward?			Giường<br>Beds
**Số lượng cũi/nôi cho trẻ sơ sinh nhập viện?**<br>Number of cots for neonatal / young infant admissions?			Cũi/nôi<br>Cots
**Số lượng sinh** tại bệnh viện hàng năm?<br>Annual number of deliveries at the hospital?			Hàng năm<br>Per year

**Số lượng nhân viên thực tế trung bình** tại **bệnh viện và tại khoa nhi** là bao nhiêu?<br>What is the **actual** (average) number of staff at the hospital and in the paediatric ward(s):	Số lượng tại bệnh viện/ các khoa phòng (Health facility (all dept.)		Số lượng tại khoa nhi<br>Paediatric dept/ward	No. of unfilled posts?
**Bác sĩ nhi khoa?**<br>Paediatricians?	*******			
**Bác sĩ?**<br>Doctors? (MOs)				
**Y sĩ?**<br>Medical assistants? (COs)				

**Y tá trực ca ngày?**<br>Nurses per day shift? (NDs)				
**Y tá trực ca đêm?**<br>Nurses during the night shift? (NNs)				
**Nhân viên hỗ trợ ca ngày?**<br>Auxiliary staff per dayshift? (ADs)				
**Nhân viên hỗ trợ ca đêm?**<br>Auxiliary staff during the nightshift? (ANs)				

**Các dịch vụ và phương tiện sẵn có: dấu x: có, để trống: không**<br>**Services and facilities available: (tick=yes, blank=no)**				
	Luôn luôn<br>Always	Hầu hết<br>Mostly	Thỉnh thoảng<br>Rarely	Không bao giờ<br>Never
**Có điện tại cơ sở y tế/bệnh viện?**<br>Do you have electricity at your health facility?	☐	☐	☐	☐
**Có nước máy?**<br>Do you have running water?	☐	☐	☐	☐
**Có xà phòng và/hoặc nước rửa tay diệt khuẩn trong khoa nhi?**<br>Do you have soap and /or disinfectant for hand-washing in the paediatric ward?	☐	☐	☐	☐
**Phòng giặt đồ và nhà vệ sinh có đủ không?**<br>Are the patient washing and toilet facilities adequate?	☐	☐	☐	☐
**Trẻ bệnh có nằm chung giường với trẻ khác không?**<br>Do children share beds with one-another?	☐	☐	☐	☐
**Có phương tiện vận chuyển giao thông sẵn có để chuyển bệnh nhân không?**<br>Is transport available to send referrals?	☐	☐	☐	☐

(UOI) |__|__|-|__|__|-|__|__| (UPI) |__|__| (UCI) |__|__|__|

**Những thiết bị dưới đây có sẵn tại bệnh viện không?**<br>**Is the following equipment available in your hospital:**					
		Always	Often	Rarely	Never
Oxy?<br>Nguồn cung cấp:	__ oxygen cylinder<br>__ oxygen concentrator<br>__ central supply	☐	☐	☐	☐
Flow mét cho oxy?<br>Flow-meters for oxygen?		☐	☐	☐	☐
**Màn hình theo dõi bão hòa oxy?**<br>Oxygen saturation monitor?		☐	☐	☐	☐
**Thiết bị quản lý oxy?**<br>Equipment for the administration of oxygen?		☐	☐	☐	☐

**Bộ kim dẫn truyền dịch dành cho nhi?**<br>IV-giving sets with chambers for paediatric use? (burette)	☐	☐	☐	☐
**Kim bướm và hoặc ống thông kích cỡ nhi?**<br>Butterflies and/or cannulas of paediatric size?	☐	☐	☐	☐
**Ống NG kích thước nhi?**<br>NG-tubes, paediatric size?	☐	☐	☐	☐
**Thiết bị quản lý dịch intra-osseous (trong xương)**<br>Equipment for intra-osseous fluid administration?	☐	☐	☐	☐
**Dụng cụ dẫn lưu?**<br>Suction equipment?	☐	☐	☐	☐
**Thiết bị phun sương cho sử dụng salbutamol?**<br>Nebulisers for administration of salbutamol?	☐	☐	☐	☐
**Buồng đệm với mặt nạ cho quản lý liều dùng Salbutamol (dạng xịt) ?**<br>Spacers with masks for administration of metered doses (spray) of salbutamol?	☐	☐	☐	☐
**Thiết bị chụp X quang chức năng?**<br>Functional X-ray equipment?	☐	☐	☐	☐

(UOI) |__|__|-|__|__|-|__|__| **(UPI) |__|__|** **(UCI) |__|__|__|**

BẢNG KIỂM QUAN SÁT
OBSERVATIONAL CHECK LIST: all inspection

Bệnh khó thở/hô hấp cấp

Breathing difficulty/Acute respiratory illness

**Look for and record presence of the following on the ward:**	**Có/Không**<br>Yes/No
**Túi chân không hoặc mặt nạ nhiều kích thước**<br>Ambu-bag and masks of different sizes?	
**Cân dùng cho trẻ sơ sinh?**<br>Scales for newborns?	
**Cân dùng cho trẻ**<br>Scales for children?	
**Máy phun khí dung?**<br>Nebulizers?	
**Buồng đệm cho quản lý sử dụng liều thuốc?**<br>Spacers for inhalation medicine metered dose administration?	
**Nhiệt kế?**<br>Thermometers?	
**Ống nghe?**<br>Stethoscopes?	

**Antiasthmatic medicine (from WHO essential drugs list):**	Yes/No
Budesonide, Inhalation (aerosol):	
3. 100 micrograms per dose;	
4. 200 micrograms per dose	
Salbutamol:	
5. Injection, 50 micrograms (as sulfate)/mL in 5-mL ampoule. 2.	
6. Metered dose inhaler (aerosol): 100 micrograms (as sulfate) per dose.	
7. Respirator solution for use in nebulizers: 5 mg (as sulfate)/mL.	
8. Oral Salbutamol	
Other inhaled cortico-steroid: ____________________________	

Other short acting beta-2 agonist: ________________________	
